# Heterostructure‐Engineered Conductive MOFs Coupled with MoSe_2_@MXene Framework for Efficient and Selective Lithium Extraction from Salt Lake Brine via Capacitive Deionization

**DOI:** 10.1002/advs.202521830

**Published:** 2026-01-04

**Authors:** Pengze Li, Xiaodan Chong, Yutong Guo, Guangrun Wu, Shanshan Wang, Ruixue Liu, Jiamiao Ma, Yu Wang, Wenqiang Chen, Yanmeng Cai, Yaoyao Wang, Qing Yuan, Jinsheng Zhao

**Affiliations:** ^1^ Shandong Key Laboratory of Chemical Energy Storage and Novel Cell Technology School of Chemistry and Chemical Engineering Liaocheng University Liaocheng China; ^2^ School of Architecture and Civil Engineering Liaocheng University Liaocheng China; ^3^ Shandong Xinfa Ruijie New Materials Technology Co., Ltd Liaocheng China

**Keywords:** capacitive deionization, c‐MOFs, heterostructure, lithium extraction, salt lake brine

## Abstract

Sustainable recovery of lithium from salt lake brines is essential for the transition toward clean energy but remains challenged by low Li^+^ concentrations and high Mg^2+^ interference. Herein, we develop a heterostructure‐engineered ternary composite, conductive metal‐organic frameworks (c‐MOFs) coupled with a MoSe_2_@MXene framework for efficient and selective lithium extraction by capacitive deionization (CDI) technology. The c‐MOFs Fe‐ tetrahydroxybenzoquinone (Fe‐THBQ) provide abundant redox‐active sites for Li⁺ capture, while the MoSe_2_@MXene framework accelerates ion transport rate and provides structural robustness. X‐ray absorption spectroscopy (XAS) confirms that strong interfacial Fe─Se coordination bonds establish a continuous Fe‐Se‐Mo charge‐transfer pathway for enhanced conductivity and ion selectivity. Thus, the Fe‐THBQ@MoSe_2_@MXene electrode delivers exceptional Li⁺ capture capacity (4.05 mmol g^−1^), ultrafast adsorption rate (0.98 mmol g^−1^ min^−1^), superior cycling stability (91.3% retention after 50 cycles), and remarkable Li⁺ selectivity with high Mg/Li ratios in both model solutions and real Dachaidan salt lake brine. Ex situ X‐ray diffraction analyses reveal a dual Li^+^ storage mechanism involving Fe^2+^/Fe^3+^ redox activity and reversible interlayer intercalation. Importantly, density functional theory (DFT) calculations confirm a substantially lower migration barrier for Li⁺ (0.052 eV) compared to competing cations, underpinning the observed selectivity. This work highlights a rational heterostructure design strategy for advancing CDI technology in lithium recovery.

## Graphical abstract

This schematic illustrates a capacitive deionization (CDI) system for the selective extraction of lithium from salt‐lake brine. The system employs a cathode fabricated from the Fe‐THBQ@MoSe_2_@MXene composite alongside an activated carbon (AC) anode. Upon applied voltage, the cathode demonstrates high efficiency in capturing Li^+^ ions while effectively excluding competing cations, thereby achieving highly selective recovery of lithium resources.

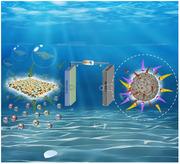

## 1 Introduction

As an important strategic resource, lithium resource plays an irreplaceable role in achieving the global goal of carbon neutrality and carbon emissions peak in the 21st century [1]. In nature, lithium occurs mainly in two forms: pegmatite‐type hard rock deposits and salt lake brines [2]. In China, roughly three‐quarters of proven lithium reserves originate from brine resources, most of which are concentrated in the salt lakes of the Qinghai‐Tibet Plateau [3]. However, extracting lithium from brine reservoirs remains technologically challenging due to the low content of Li^+^ and the complex coexistence of competing cations. In particular, the nearly identical ionic radii of Li^+^ and Mg^2+^ create difficulties in selective separation, while the dilute concentrations of lithium further reduce recovery efficiency [4, 5, 6]. These limitations translate into high energy requirements, high operational difficulties, and poor yields when using conventional recovery strategies such as chemical precipitation or adsorption‐based methods [7]. Therefore, there is a pressing need to develop green and efficient new lithium extraction technology [8]. Capacitive deionization (CDI) technology has become a promising approach for lithium extraction for its unique advantages, such as low energy consumption, high adsorption selectivity, and applicability to complex solution systems [9]. The underlying mechanism relies on either the electric double layer or pseudocapacitive behavior. When an external electric field (≤ 1.2 V) is applied, Li^+^ are preferentially immobilized on the electrode through electrosorption or lattice intercalation, while anions migrate toward the counter electrode to maintain charge balance [10]. Upon reversing the applied voltage, the trapped ions are released, enabling both electrode regeneration and recovery of lithium resources [11].

The key to efficient and selective extraction of Li^+^ by CDI is to utilize functional materials with specific recognition for Li^+^ [12]. The commonly adopted Li^+^ capture materials include LiFePO_4_ (LFP) and LiMn_2_O_4_ (LMO). LFP has a unique olivine‐type structure with a suitable channel size with the radius of Li^+^ (0.076 nm), which can form a size‐screening effect to prevent the insertion of larger ions like Na^+^ and Mg^2+^, facilitating Li^+^ transport [13]. However, LFP is limited by its low conductivity and theoretical specific capacity, leading to slow adsorption kinetics. LMO features a spinel‐type crystal structure, which permits Li^+^ to move freely in spatial dimensions and promote rapid ionic transport [14]. But the poor cycle stability of LMO due to its lattice distortion caused by the Jahn‐Teller effect has restricted its further application in lithium extraction [15]. Thus, developing new materials with high capacity, excellent cycle stability, and high Li^+^ selectivity has become an urgent research focus.

Conductive metal‐organic frameworks (c‐MOFs), a new class of crystalline porous materials, exhibit unique structural advantages and functional properties through the directional coordination self‐assembly of metal ions/clusters and organic ligands [16]. Due to its ultra‐high specific surface area, tunable pore size distribution, and inherent pseudocapacitive characteristics, it shows significant advantages in electrochemical energy storage. Jiang et al. reported a Cu‐based c‐MOFs (Cu‐THQ) as a cathode material for lithium‐ion batteries, which achieved a high capacity of 387 mAh g^−1^ and an energy density of 775 Wh kg^−1^ [17]. Li et al. reported a La‐based amino‐functionalized MOF (LaBDC/C‐NH_2_) as an anode material for CDI, achieving a high F^−^ adsorption capacity of 73.8 mg g^−1^ and a remarkable F^−^/Cl^−^ selectivity factor of 56.52 through a synergistic mechanism of electrostatic attraction, amino group protonation, and ion exchange with hydroxyl groups [18]. However, the self‐stacking tendency of c‐MOFs due to the uncontrolled crystal growth impedes the accessibility of active sites, leading to low Li^+^ extraction efficiency. Cooperating c‐MOFs with an appropriate substrate can be regarded as a feasible coordination control strategy to precisely adjust the crystal growth direction of c‐MOFs. Traditional carbonaceous substrates (such as graphene, carbon cloth, and carbon nanotubes) can provide good conductive support. Feng et al. anchored MOF nanoparticles uniformly on a 3D porous graphene framework (HG) and constructed a MOF/HG composite, which has high specific capacitance (526 F g^−1^ at a current density of 0.1 A g^−1^) and considerable electrosorption capacity (39.6 mg g^−1^ in 800 mg L^−1^ NaCl solution) [19]. Geng et al. further advanced this concept by developing a surface coordination control strategy guided by a carbon substrate to achieve anisotropic morphology in Fe‐MOFs. The oxygen‐containing functional groups on the carbon surface selectively coordinated with unsaturated Fe^3+^ sites on the [111] crystal plane, effectively suppressing growth along the <111> orientation. This morphology regulation enhanced the Li^+^ storage rate performance, with a capacity retention of 81.1% at 1000 mA g^−1^ [20]. Nevertheless, carbon‐based substrates have intrinsic limitations: their ion storage capability is restricted to physical adsorption via electric double‐layer capacitance, and they lack Li^+^‐specific recognition sites, making it difficult to achieve enhanced selectivity through synergistic effects.

To solve these problems, pseudocapacitive heterostructure engineering has emerged as an innovative strategy. Specifically, the pseudocapacitive heterostructure MoSe_2_@MXene has been proven to have enhanced conductivity, promoted charge‐transfer kinetics, and improved structural durability due to the synergistic effect of MoSe_2_ and MXene [21]. In the heterostructure MoSe_2_@MXene, the abundant surface functional groups (such as ─O, ─F) of MXene can form coordination bonds with c‐MOFs to achieve uniform dispersion. MoSe_2_ can efficiently trap and store Li^+^ through the unique intercalation mechanism for its moderate interlayer spacing. Meanwhile, the tunable valence band structure of 1.1–1.5 eV endows it with excellent charge storage characteristics [22]. The synergistic coupling of c‐MOFs with MoSe_2_@MXene can build a 3D conductive network, which can effectively regulate the growth direction of crystal planes and expose active sites while inhibiting the self‐stacking of c‐MOFs, thus significantly improving the lithium extraction and selectivity performance.

In this study, we successfully constructed a novel ternary composite by combining Fe‐tetrahydroxybenzoquinone (Fe‐THBQ) with MoSe_2_@MXene pseudocapacitive heterostructure. In this system, Fe‐THBQ acts as a Li^+^ trapping layer to achieve efficient capture through its abundant redox active sites. Meanwhile, the MoSe_2_@MXene heterostructure provides a 3D fast ion transport channel. The synergistic effect of Fe‐THBQ and MoSe_2_@MXene not only significantly increases the accessible active sites but also optimizes the ion transport kinetics, thus realizing the efficient capture of Li^+^. X‐ray absorption spectroscopy (XAS) was used to characterize the coordination environment of Fe in the composite, and the existence of an Fe─Se bond confirmed the chemical bonding between Fe‐THBQ and MoSe_2_@MXene, which not only enhanced the structural stability of the material, but also constructed a continuous Fe‐Se‐Mo charge conduction channel. The electrochemical performance and lithium extraction performance of the ternary composite were systematically tested. The results showed that Fe‐THBQ@MoSe_2_@MXene exhibited high specific capacitance and adsorption capacity. The Li^+^ insertion/extraction in the ternary composite through the Fe‐THBQ reversible redox reaction and MoSe_2_ reversible intercalation reaction was analyzed by ex situ XRD. Density functional theory calculations illustrate the intrinsic mechanism of Li^+^ selectivity. The migration energy barrier of Li^+^ in Fe‐THBQ@MoSe_2_@MXene is only 0.052 eV, which is much lower than that of other cations. This precise heterogeneous interface design and multi‐component synergy provide a new material system for the development of efficient lithium resource mining.

## 2 Results and Discussion

### 2.1 Synthesis of Ternary Composite and Material Characterization

**SCHEME 1:**
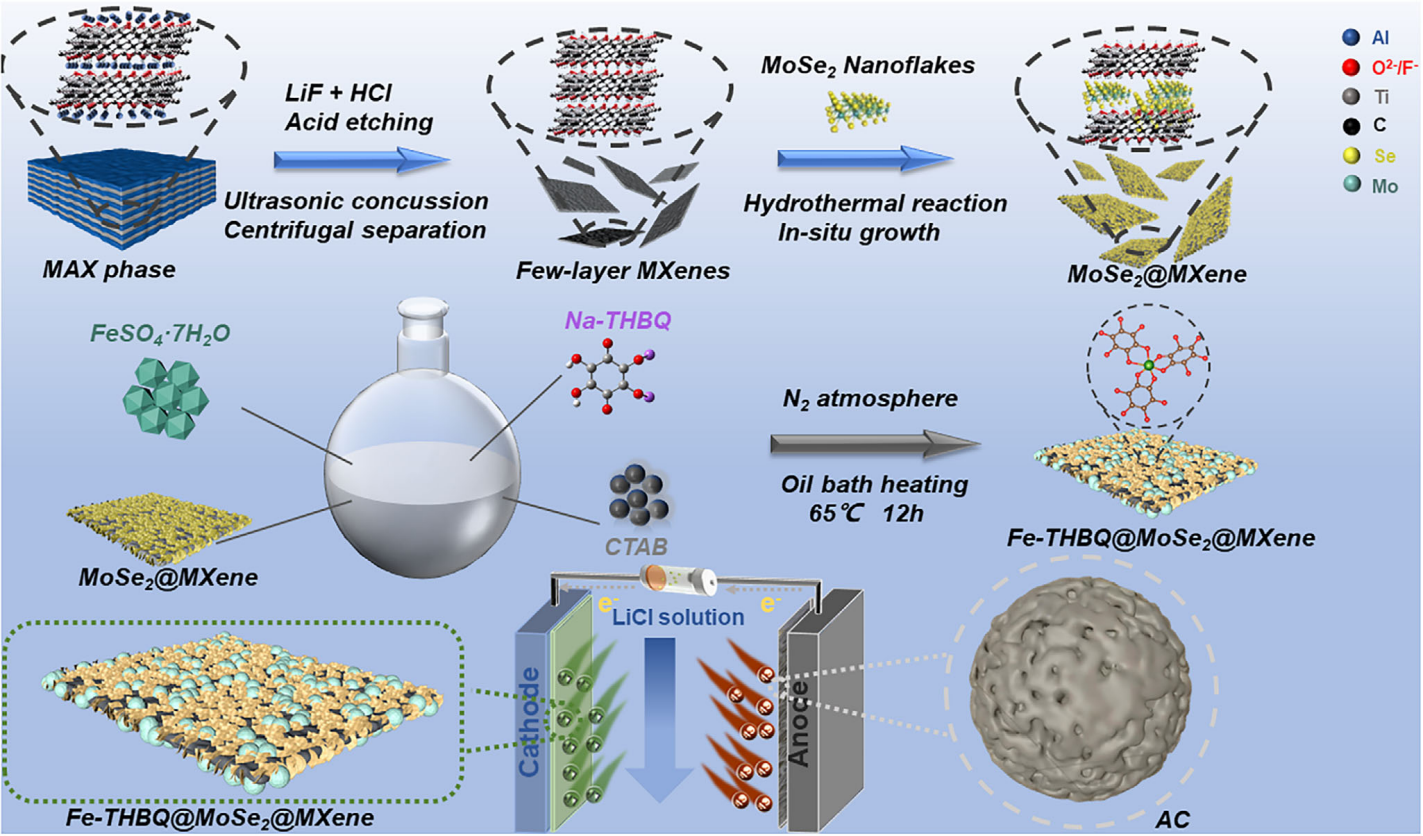
Schematic diagram of the synthesis process of ternary material Fe‐THBQ@MoSe_2_@MXene.

The synthesis process of ternary composite Fe‐THBQ@MoSe_2_@MXene is shown in Scheme 1. First, few‐layered MXene nanosheets were obtained by acid etching and ultrasonic exfoliation of titanium aluminum carbide (Ti_3_AlC_2_) as a structurally stable conductive substrate and a growth matrix rich in functional groups. Subsequently, MoSe_2_ was vertically grown on its surface by the hydrothermal method to form a binary heterostructure, MoSe_2_@MXene. On this basis, Fe‐THBQ was uniformly loaded on the surface of MoSe_2_@MXene by an in situ growth strategy, and the ternary composite Fe‐THBQ@MoSe_2_@MXene was successfully constructed. It is noteworthy that MXene sheets not only provide a vertically growing conductive framework for MoSe_2_ nanosheets but also effectively inhibit the agglomeration of Fe‐THBQ. Fe‐THBQ not only retains its inherent high specific surface area and rich pore structure, but also has a synergistic effect with MoSe_2_@MXene in the inner layer, which significantly improves the charge transport efficiency and structural stability of the composite. This unique ternary heterostructure not only accelerates ion transport kinetics but also increases accessible active sites for Li^+^.

**FIGURE 1:**
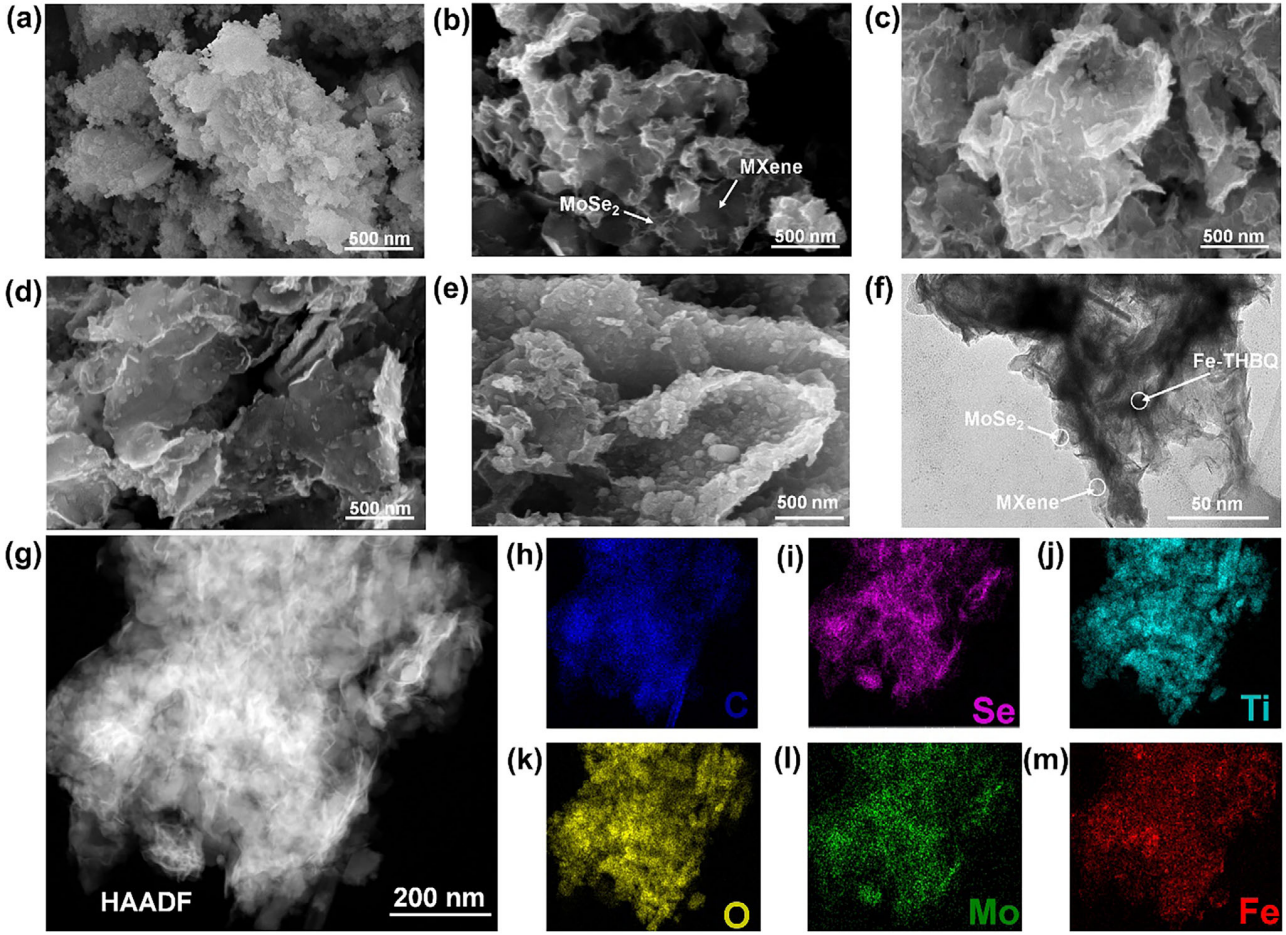
SEM images of a) Fe‐THBQ, b) MoSe_2_@MXene, and c–e) Fe‐THBQ@MoSe_2_@MXene‐x (x = 0.5, 1, 1.5). f) TEM and g) HAADF of Fe‐THBQ@MoSe_2_@MXene‐1. The corresponding mapping images of C h), Se i), Ti j), O k), Mo l), and Fe m) elements for Fe‐THBQ@MoSe_2_@MXene‐1.

The morphologies of all materials were analyzed by scanning electron microscopy (SEM) and transmission electron microscopy (TEM). In Figure 1a, Fe‐THBQ aggregates into irregularly‐shaped clusters due to its high surface energy and non‐oriented facet growth. Compared Figure 1b with Figure S3a,b, it can be found that MoSe_2_ nanosheets are successfully anchored on the smooth MXene surface, forming a roughened 3D structure, MoSe_2_@MXene. Through the systematic analysis of ternary materials with different proportions, the structural evolution process can be clearly observed. When the Fe‐THBQ loading is low (Figure 1c,d), Fe‐THBQ particles can be uniformly dispersed on the surface of the MoSe_2_@MXene heterostructure. In contrast, when the Fe‐THBQ loading is increased to an excess level (Figure 1e), as the growth sites on the surface of the MoSe_2_@MXene framework tend to be saturated and the excess Fe‐THBQ tends to locally self‐aggregate together, which is likely to block the open mesoporous channels of Fe‐THBQ itself and impede ion transport kinetics, leading to performance degradation. The TEM image and HRTEM image (Figure 1f; Figure S3c) also confirm that Fe‐THBQ@MoSe_2_@MXene‐1 possesses a 3D sheet‐like cross‐linked architecture with Fe‐THBQ uniformly anchored at the heterointerfaces. Furthermore, the HAADF image (Figure 1g) together with the corresponding elemental mapping results (Figure 1h–m) demonstrates the homogeneous distribution of C, Ti, O, Mo, Se, and Fe throughout the ternary composite, indicating the uniform loading of MoSe_2_ and Fe‐THBQ onto the MXene surface.

**FIGURE 2:**
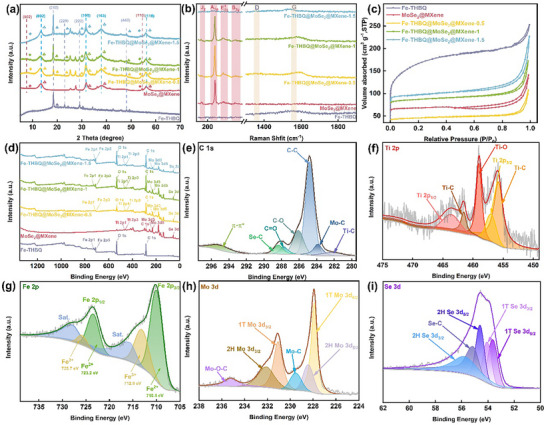
(a) XRD patterns, b) Raman spectra, c) N_2_ adsorption‐desorption isotherms, and (d) XPS survey spectra of all samples. High‐resolution XPS spectra of Fe‐THBQ@MoSe_2_@MXene‐1: e) C 1s, f) Ti 2p, g) Fe 2p, h) Mo 3d, and i) Se 3d.

The phase structure and crystal structure of the prepared materials were analyzed by X‐ray diffraction (XRD) and Raman spectroscopy. Figure 2a shows that across all ternary composites, the characteristic diffraction peaks of Fe‐THBQ at 18.5°, 23.1°, 29.1°, and 48.2° are fully preserved, and the peak intensity increases with higher Fe‐THBQ loading, indicating that the crystal structure of Fe‐THBQ remains unchanged during the composite formation and further confirming the controllability of the quantitative composite process. Meanwhile, as the Fe‐THBQ loading increases, the intensity of the characteristic peaks for MoSe_2_ and MXene gradually decreases, while their peak positions remain unchanged. This stability in peak position indicates that the framework structure of the MoSe_2_@MXene is fully retained during the compositing process [20, 24] The Raman spectra (Figure 2b) of MoSe_2_@MXene and Fe‐THBQ@MoSe_2_@MXene‐x (*x* = 0.5, 1, and 1.5) show the characteristic peaks of A_1g_ (239 cm^−1^), E^1^_2g_ (284 cm^−1^), B_2g_ (339 cm^−1^). They are all assigned to 2H‐MoSe_2_, and it can be noted that with the increase of Fe‐THBQ loading, the peak intensity gradually decreases, which can be attributed to the limitation of the MoSe_2_ surface vibration mode caused by Fe‐THBQ coverage, supporting the conclusion in the XRD spectrum. In addition, an additional stretching resonance peak at 152 cm^−1^ can be identified as the J_2_ peak of 1T‐MoSe_2_ 23[25, 26]. 1T phase promotes charge transport, 2H phase provides stable active sites, and the existence of a hybrid phase will cooperate to optimize the overall performance of the composite. The broad diffraction peaks at 1355 and 1573 cm^−1^ for the ternary composite are associated with the sp_3_ hybridized D band and the sp_2_ hybridized G band of the Fe‐THBQ, respectively, reflecting the degree of defects and graphitization of the material [27, 28].

The specific surface area (SSA) and pore size distribution of all the prepared materials were systematically evaluated by N_2_ adsorption‐desorption isotherms (Figure 2c). Fe‐THBQ showed the highest SSA (232.71 m^2^ g^−1^), and the SSA of Fe‐THBQ@MoSe_2_@MXene‐x (*x* = 0.5, 1, and 1.5) showed a regular growth trend by increasing Fe‐THBQ loading with the values of 87.34, 153.26, and 192.45 m^2^ g^−1^ respectively, which are significantly higher than the SSA of MoSe_2_@MXene (59.98 m^2^ g^−1^), indicating that the Fe‐THBQ plays a beneficial role in improving the SSA of the composite. The Barrett‐Joyner‐Halenda (BJH) pore distribution analysis (Figure S4) reveals Fe‐THBQ shows typical microporous characteristics, while the MoSe_2_@MXene heterostructure is dominated by the mesoporous structure. By increasing Fe‐THBQ loading, the pore size distribution of the ternary composites showed an obvious trend of increasing microporosity. This precisely controlled pore size distribution change confirms the successful grafting of Fe‐THBQ. The increased SSA and microporous properties are expected to provide more abundant active sites and efficient transport channels [29, 30, 31].

X‐ray photoelectron spectroscopy (XPS) was used to analyze the elemental composition, chemical state, and interfacial interaction of the materials. XPS full spectrum (Figure 2d) shows that C, O, Fe, Ti, Mo, and Se elements are present in the ternary composite. C 1s high resolution spectrum of Fe‐THBQ@MoSe_2_@MXene‐1 (Figure 2e) shows seven characteristic peaks at 282.1, 283.9, 284.8, 286.1, 287.7, 288.4, and 295.1 eV, corresponding to Ti─C, Mo─C, C─C, C─O, C═O, Se─C, *π*–*π* ^*^ bonds. The characteristic peak of the Ti─C bond is derived from the MXene substrate. The appearance of Mo─C and Se─C bonds indicates the formation of strong covalent interfacial connections between MoSe_2_ and the interfaces of both Fe‐THBQ and MXene [32]. These covalent bonds not only significantly enhance the electronic coupling effect between the components but also effectively inhibit the expansion or dissolution of the material by building a stable chemical bond network. The Ti 2p high‐resolution XPS spectrum (Figure 2f) shows typical spin‐orbit splitting characteristics, in which the characteristic peaks at 457.1 and 463.7 eV correspond to the Ti 2p_3/2_ and Ti 2p_1/2_ levels, respectively. The double peaks at 455.8 and 456.1 eV correspond to Ti‐C 2p_3/2_ and 2p_1/2_ orbitals, confirming the intact Ti─C bonding in MXene. The prominent peak at 459.1 eV is attributed to Ti─O bonds originating from oxygen‐containing groups introduced during material synthesis [33]. O 1s (Figure S5) revealed three oxygen species: lattice oxygen (530.4 eV, Fe─O bond), hydroxyl oxygen (532.2 eV, adsorbed H_2_O), and adsorbed oxygen (533.4 eV, C═O) [34]. The Fe 2p spectrum (Figure 2g) shows that peaks at 725.7, 723.2, 712.9, and 710.1 eV can be assigned to Fe^3+^ 2p_1/2_, Fe^2+^ 2p_1/2_, Fe^3+^ 2p_3/2,_ and Fe^2+^ 2p_3/2_, respectively [35, 36, 37]. Mo 3d spectrum (Figure 2h) shows double peaks of 1T phase at 227.9 eV (3d_5/2_) and 231.1 eV (3d_3/2_), and 2H phase at 228.4 eV (3d_5/2_) and 232.1 eV (3d_3/2_), indicating the coexistence of 1T and 2H phases. The Mo─C bond at 229.5 eV confirms the formation of a covalent bond between MoSe_2_ and MXene, providing a stable heterostructure. In particular, the Mo─O─C bond at 235.2 eV indicates that there is an oxygen‐bridged interfacial connection, which effectively buffers the volume change during the electrochemical process and provides efficient transport channels for charge transfer [38, 39, 40]. Se 3d spectrum (Figure 2i) shows two pairs of characteristic peaks corresponding to 1T MoSe_2_ (53.6 eV for Se 3d_5/2_ and 54.1 eV for Se 3d_3/2_), 2H MoSe_2_ (Se 3d_5/2_ is 54.6 eV and Se 3d_3/2_ is 55.8 eV). This is in high agreement with the Mo 3d spectra, confirming the coexistence of the 1T phase with the 2H phase. The Se─C bond (55.2 eV) further indicates that MoSe_2_ forms a covalent bond with the carbon matrix, which is beneficial for enhancing the structural stability [41].

**FIGURE 3:**
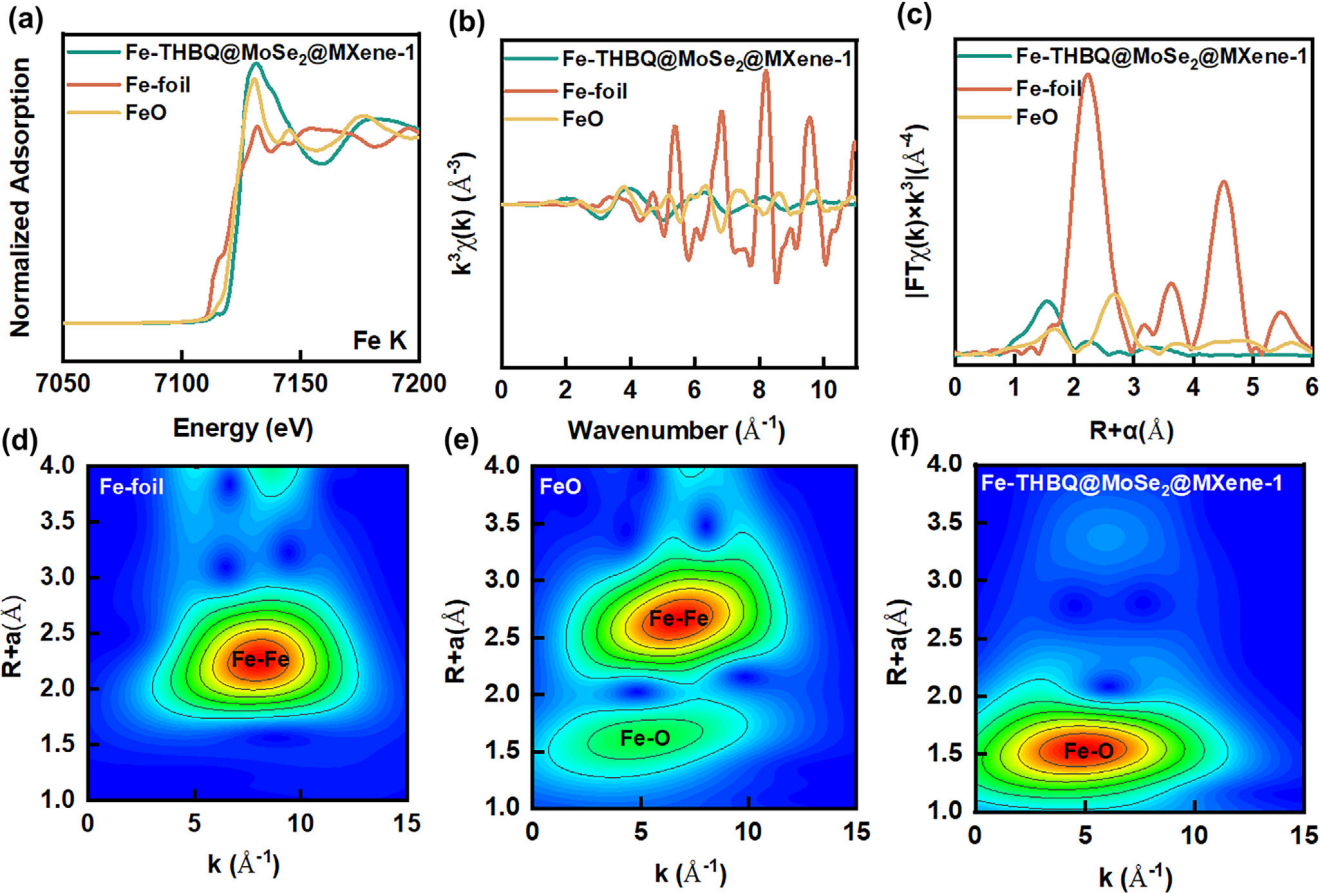
(a) Normalized Fe K‐edge X‐ray near absorption near edge spectra of Fe‐foil, FeO, and Fe‐THBQ@MoSe_2_@MXene‐1. The corresponding Fe K‐edge Extended X‐ray Absorption Fine Structure (EXAFS) is shown in k_3_ weighted b) k‐space and c) R‐space. Wavelet transform of k_3_ weighted EXAFS signal of Fe‐foil d), FeO e), and Fe‐THBQ@MoSe_2_@MXene‐1 f) using Morlet wavelet with κ = 10, σ = 1.

The Fe K‐edge X‐ray absorption near‐edge structure (XANES) spectra (Figure 3a) show that the absorption edge position of Fe‐THBQ@MoSe_2_@MXene‐1 is close to the standard reference of FeO (Fe^2+^) and shows a slight shift to the high‐energy direction, which confirms that the main chemical state of Fe is +2 valence. Meanwhile, there is a slight oxidation (partial Fe^2+^ → Fe^3+^) during the recombination process, revealing the electronic structure evolution and surface oxidation behavior of Fe. The pre‐edge peak of the composite is absent, and the white line peak is enhanced and broadened is observed, indicating that the original coordination configuration of Fe is still retained after the deposition of Fe‐THBQ on the MoSe_2_@MXene, ensuring the intrinsic activity of the Fe redox center in the ternary composite. The enhanced white‐line peak indicates strong orbital hybridization, which promotes charge delocalization and significantly improves electronic conductivity [42, 43, 44].

Extended X‐ray absorption fine structure (EXAFS) spectroscopy (Figure 3b) shows that the oscillation amplitude of the composite is highly consistent with that of FeO, which confirms that the Fe─O coordination bond is dominant in the composite. This finding is consistent with the valence characteristics of Fe^2+^ in XANES, indicating that the composite successfully retains the basic coordination framework of Fe‐THBQ. It is worth noting that compared with FeO, the composite shows a lower oscillation frequency, which directly reflects the increase of Fe─O bond length, which may be due to the strain at the MoSe_2_@MXene heterointerface during the recombination process. The increase in bond length will weaken the orbital overlap between the metal center (Fe) and the ligand (O), enhance the delocalization of electrons, and improve the efficiency of charge transport. Meanwhile, longer bonds usually weaken the Fe‐O interaction, which may reduce the adsorption energy barrier and improve the kinetics. The composites exhibit faster amplitude decay in the K‐space spectra, indicating that the degree of local structural disorder increases significantly, and this change in structural characteristics is beneficial to reduce electron localization and improve ion migration kinetics. The R‐space spectra after Fourier transform (Figure 3c) show that the composite exhibits a significant characteristic peak at 1.55 Å, which corresponds to the first coordination shell of Fe. Compared with the standard sample FeO, the peak position of the composite is shifted to the low R direction by about 0.12 Å, which proves that the Fe─O bond length has increased. A new coordination peak appears in the range of 2.3–2.5 Å, which is highly consistent with the theoretical prediction of Fe─Se bond length (≈ 2.4 Å), confirming that Fe and Se are chemically bonded at the heterointerface, creating a built‐in electronic path through Fe‐Se‐Mo bridging and effectively reducing the interface contact resistance. The scattering intensity of the second coordination shell (3.0–3.5 Å) is significantly reduced compared with the FeO and Fe‐foil standard samples, indicating that the long‐range order of the composite is significantly destroyed [45, 46].

Wavelet transform (Figure 3d–f) is equivalent to the complementary analysis of R‐space, which provides finer atomic‐scale information for the local structure of composite materials through the simultaneous analysis of K‐space oscillation characteristics and R‐space distance information. In K‐space, the decrease of the oscillation frequency of the composite indicates the increase of the Fe─O bond length, and the wavelet transform further distinguishes the contributions of different mass coordination atoms through the scale‐position analysis: the low‐frequency component corresponds to the heavy atom coordination, such as Fe‐Se, and the high‐frequency component reflects the light atom interaction, such as Fe‐O. Compared with the overlap signal of the main peak at 2.09 Å and the secondary peak at 3.25 Å in the traditional R‐space spectrum, the Fe‐Se coordination feature in the range of 2.3–2.5 Å was clearly resolved by wavelet transform, and the superposition contribution of multiple scattering effect in the range of 3.0–3.5 Å was identified. This multi‐scale analysis finally confirmed qualitatively that Fe was coordinated with both O and Se atoms in the composite, and the spatial asymmetry of the O/Se ligand field creates diversified adsorption sites; the Fe─O polar bond maintains the redox activity of Fe^2+^, while the Fe─Se metallic bond realizes long‐range electron delocalization. This synergistic effect of electronic state regulation and interfacial charge delocalization is expected to achieve the simultaneous improvement of Li^+^ capture capacity [47].

### 2.2 Electrochemical Performance

**FIGURE 4:**
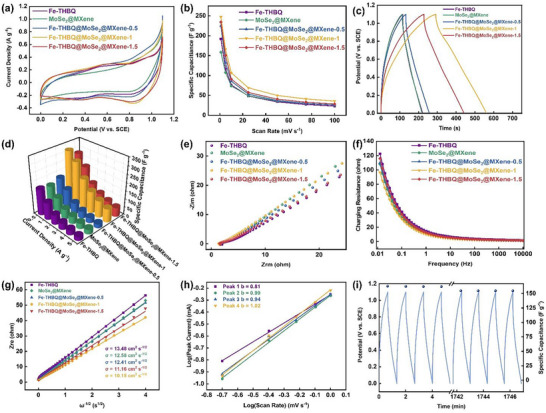
(a) CV curves in 1 m LiCl solutions at 1 mV s^−1^ within the potential window of 0–1.1 V. b) Specific capacitance (F g^−1^) with respect to the scan rate (1–100 mV s^−1^). c) GCD curves in 1 m LiCl solutions at 0.5 A g^−1^. d) Specific capacitance (F g^−1^) with respect to the current density (0.5–5 A g^−1^). e) Nyquist plots. f) Charging resistances. g) The relationship between the Z_re_ (ohm) and the angular frequency ω^−1/2^ (s^1/2^). h) The relationship between the peak current (mA) and scan rate (mV s^−1^). i) The specific capacitance of the Fe‐THBQ@MoSe_2_@MXene‐1 electrode during 1500 GCD cycles.

The electrochemical performance of the five electrode materials was systematically evaluated in 1 M LiCl electrolyte using cyclic voltammetry (CV), electrochemical impedance spectroscopy (EIS), and galvanostatic charge‐discharge (GCD) measurements. In Figure 4a, the CV curve of the Fe‐THBQ electrode exhibits distinct redox peaks, attributed to the faradaic reaction of the Fe^2+^/Fe^3+^ redox couple. MoSe_2_@MXene exhibits a quasi‐rectangular CV curve, which is due to the pseudocapacitive contribution of the intercalation. The CV curves of the three ternary composites (Fe‐THBQ@MoSe_2_@MXene) exhibit significantly different electrochemical characteristics from those of the single component and binary systems [48, 49]. As the Fe‐THBQ loading increases, the CV curves show progressively enhanced redox peaks. This transition indicates a fundamental shift in the lithium storage mechanism from an intercalation behavior to a redox process. It is worth noting that the significant increase in the integral area of the CV curve directly reflects the significant improvement in the specific capacitance [50]. Moreover, the CV curve characteristics of the five electrodes at different scan rates (1–100 mV s^−1^) were well maintained, reflecting their excellent electrochemical reversibility (Figure S6). In Figure 4b, the specific capacitance of all electrodes decreases with increasing scanning rate, which can be attributed to the fact that Li^+^ in the electrolyte cannot diffuse to the deep position of the material at a higher scanning rate, resulting in the invalid active sites, and the surface redox reaction cannot be fully carried out [51]. Obviously, the Fe‐THBQ@MoSe_2_@MXene‐1 electrode shows the highest capacity performance at all scan rates, which benefits from the unique 3D charge transport network of Fe‐THBQ and MoSe_2_@MXene, greatly reducing the charge transfer resistance and shortening the charge transport path. It is noteworthy that when the theoretical loading of Fe‐THBQ exceeds the critical value of 1:1 relative to MoSe_2_@MXene, its electrochemical performance will be significantly reduced. This is primarily because surplus Fe‐THBQ can block the mesoporous channels within the MoSe_2_@MXene heterostructure, disrupt the original 3D conductive network, hinder electrolyte penetration, and impede charge transport kinetics.

In Figure 4c, the discharge time of Fe‐THBQ@MoSe_2_@MXene‐1 electrode is much longer than that of other electrodes, showing excellent electrochemical energy storage performance. Under the test condition of 1 A g^−1^, the specific capacitance of the electrode reaches 273.2 F g^−1^, which is 2.56 and 2.58 times that of Fe‐THBQ (106.6 F g^−1^) and MoSe_2_@MXene (105.8 F g^−1^) electrodes, respectively. This marked enhancement in performance can be attributed to the material's innovative structural design. MXene, as a conductive framework, ensures the fast conduction of electrons. The layered structure of MoSe_2_ provides fast ion transport channels. The uniform distribution of Fe‐THBQ contributes numerous redox‐active sites. The three components cooperate to build an efficient charge transport network. This advantage is maintained at different current densities (0.5–5 A g^−1^) (Figure 4d), which fully demonstrates the rationality and practicability of the material design.

Electrochemical impedance spectroscopy (EIS) was performed to investigate their charge‐transfer kinetics and ion diffusion behavior. In Figure 4e, the Nyquist diagram consists of a semicircular arc in the high frequency region and a straight line in the low frequency region. The smaller the semicircular arc is, the smaller the charge transfer resistance (R_ct_) is, and the easier it is for electrons to pass through the surface of the electrode material, while the larger the slope of the oblique line is, the faster the ion diffusion rate is. R_ct_ values of Fe‐THBQ, MoSe_2_@MXene, Fe‐THBQ@MoSe_2_@MXene‐0.5, Fe‐THBQ@MoSe_2_@MXene‐1, Fe‐THBQ@MoSe_2_@MXene‐1.5 are 2.31, 1.50, 1.44, 0.97, and 1.72 Ω, respectively. The Fe‐THBQ@MoSe_2_@MXene‐1 electrode exhibits the lowest R_ct_ across the entire frequency range (Figure 4f), indicating its superior structural configuration. The Fe‐THBQ electrode shows the smallest Warburg slope in the low frequency region, indicating that their ion diffusion rate is the slowest, which is mainly due to their severe stacking, resulting in great resistance to Li^+^ migration. With the introduction of MoSe_2_@MXene, the ternary composite exhibits significantly increased slopes, which is due to the fact that the 3D mesoporous channel constructed by MXene significantly improves the ion transport path [52, 53]. Fe‐THBQ@MoSe_2_@MXene‐1 electrode has the largest slope in the low frequency region for its exquisite material design and optimal ion diffusion kinetics. This behavior is fully consistent with the findings from CV and GCD analyses.

In order to understand the ion diffusion kinetics, the ion diffusion coefficient (D) is used for quantitative analysis, and the corresponding expression is as follows:

(1) $$Z_{re}=\sigma \cdot \omega^{-1/2}$$

(2) $$D=\frac{R^{2}T^{2}}{2A^{2}n^{4}F^{4}C^{2}\sigma^{2}}$$

where Z_re_, σ, ω, R, T, A, n, F, C represent the real part of the impedance, the Warburg coefficient, the angular frequency, the ideal gas constant, the electrolyte temperature, the area of electrode sheet, the number of electrons transferred, the Faraday constant, and the electrolyte concentration, respectively. The σ value of Fe‐THBQ@MoSe_2_@MXene‐1 electrode (10.15) is lower than that of Fe‐THBQ (13.48), MoSe_2_@MXene (11.16), Fe‐THBQ@MoSe_2_@MXene‐0.5 (12.41), and Fe‐THBQ@MoSe_2_@MXene‐1.5 (12.58) electrodes (Figure 4g). The calculated D follows the order: Fe‐THBQ@MoSe_2_@MXene‐1 (3.21×10^−17^ cm^2^ s^−1^) > MoSe_2_@MXene (2.03×10^−17^ cm^2^ s^−1^) > Fe‐THBQ@MoSe_2_@MXene‐1.5 (1.72×10^−17^ cm^2^ s^−1^) > Fe‐THBQ@MoSe_2_@MXene‐0.5 (1.85×10^−17^ cm^2^ s^−1^) > Fe‐THBQ (1.12×10^−18^ cm^2^ s^−1^). The diffusion rate of Li^+^ can be enhanced by an order of magnitude when the mass ratio of Fe‐THBQ, MoSe_2_, and MXene reaches the optimal ratio.

The electrochemical kinetics of the Fe‐THBQ@MoSe_2_@MXene‐1 electrode were further clarified by analyzing CV curves at different scan rates (Figure S7). The relationship between scan rate (v) and peak current (i) was expressed by a logarithmic equation [54]:

(3) logi=blogv+loga

The b values of peaks 1–4 in Figure 4h are 0.81, 0.99, 0.94, and 1.02, respectively, indicating that capacitive behavior predominates in the Li⁺ storage process of the Fe‐THBQ@MoSe_2_@MXene‐1 electrode.

In electrochemical energy storage systems, the current response in CV measurements typically comprises contributions from two processes: capacitive control (k_1_v) and diffusion‐controlled behavior (k_2_v^1/2^), and its quantitative relationship can be expressed as:

(4) $$i=k_{1}v+k_{2}v^{1/2}$$

where i represents the peak current (A), v is the scan rate (mV s^−1^), and k_1_ and k_2_ are proportional constants characterizing the contribution of the capacitive behavior and diffusion‐controlled process, respectively. Obviously, as the scan rate increases from 0.2 to 1.0 mV s^−1^, the capacitive contribution of the Fe‐THBQ@MoSe_2_@MXene‐1 electrode rises markedly from 74% to 86%. (Figure S8). This phenomenon clearly confirms that with the increase of the scan rate, the energy storage process of the electrode is gradually dominated by the surface‐controlled fast redox reaction. This dynamic behavior change is mainly due to the following two factors: on the one hand, Fe‐THBQ provides abundant electrochemically active sites, which greatly promote the rapid Faradaic reaction of Li^+^; on the other hand, the well‐constructed 3D conductive network in the material significantly improves the efficiency of electron conduction and provides an efficient charge transport channel for redox reactions. These structural advantages together ensure that the electrode can maintain excellent electrochemical performance at high scan rates.

The Fe‐THBQ@MoSe_2_@MXene‐1 electrode maintains 95% capacity retention after 1500 GCD cycles at a high current density of 4 A g^−1^ (Figure 4i), demonstrating its excellent cycling stability. This excellent cycle performance can be attributed to the fact that the heterostructure MoSe_2_@MXene inhibits the structural degradation caused by the dissolution of the active material during cycling. Meanwhile, the Fe‐THBQ framework can withstand large mechanical stress through its strong coordination network and porous structure. More importantly, the unique flexible 3D structure can effectively buffer the volumetric strain caused by repeated intercalation/deintercalation of lithium ions, thus maintaining the integrity of the electrode structure. These structural advantages together ensure the stability and reversibility of the electrode in the long‐term cycle process.

### 2.3 CDI Performance

**FIGURE 5:**
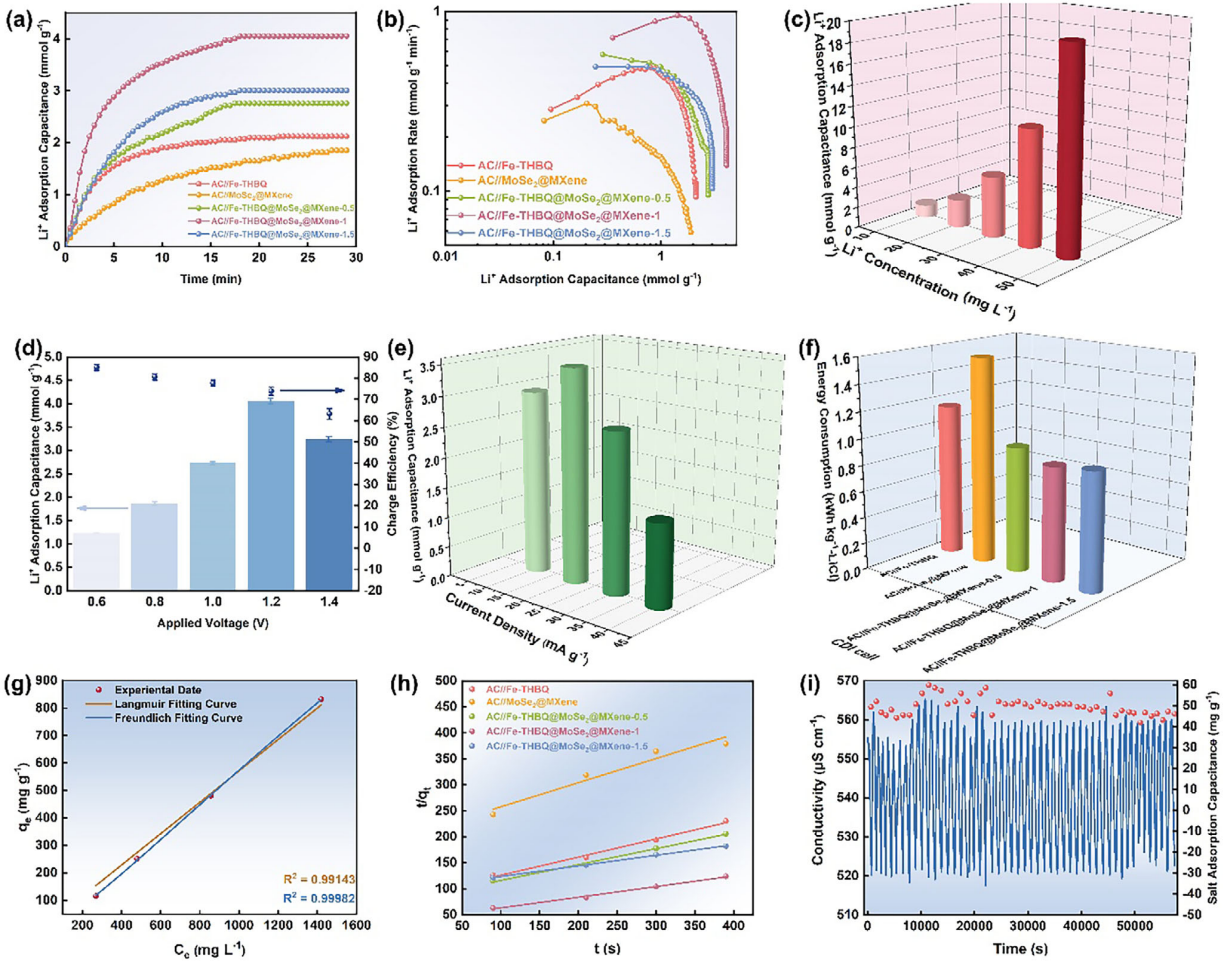
(a) The Li^+^ adsorption capacity varies over time. b) Ragone plots. c) The Li^+^ adsorption capacity for AC//Fe‐THBQ@MoSe_2_@MXene‐1 cell under different Li^+^ concentrations. d) The Li^+^ adsorption capacity for the AC//Fe‐THBQ@MoSe_2_@MXene‐1 cell under different applied voltages. e) The Li^+^ adsorption capacity for the AC//Fe‐THBQ@MoSe_2_@MXene‐1 cell under different current densities. f) The corresponding energy consumption in 10 mmol L^−1^ LiCl solution at 1.2 V. g) The Langmuir and Freundlich isotherm model for the AC//Fe‐THBQ@MoSe_2_@MXene‐1 cell. h) The pseudo‐second‐order kinetic fitting curves. i) 50 cycles of the AC//Fe‐THBQ@MoSe_2_@MXene‐1 cell in 5 mmol L^−1^ LiCl solution.

Asymmetric CDI cells were assembled using the prepared electrodes as the cathode and activated carbon (AC) as the anode to assess their Li^+^ extraction capacity. Figure 5a shows the Li^+^ adsorption curves of five CDI cells in 10 mmol L^−1^ LiCl solution at 1.2 V. In the initial stage, the adsorption capacity increased rapidly, indicating the rapid enrichment of Li^+^ and Cl^−^ on the electrode surface driven by the electric field. With the passage of time, the adsorption curve tended to be flat, indicating that the electrode reached a saturated adsorption equilibrium state [55]. Obviously, the AC//Fe‐THBQ@MoSe_2_@MXene‐1 cell exhibits the optimal Li^+^ capture ability, with a maximum adsorption capacity of 4.05 mmol g^−1^, which is significantly higher than that of AC//Fe‐THBQ (2.12 mmol g^−1^) and AC//MoSe_2_@MXene (1.85 mmol g^−1^) cells. In addition, the AC//Fe‐THBQ@MoSe_2_@MXene‐1 curve is located in the upper‐rightmost region of the Ragone plot (Figure 5b), illustrating the fastest Li^+^ adsorption rate (0.98 mmol g^−1^ min^−1^). Meanwhile, Figure 5f and Figure S9 show that the AC//Fe‐THBQ@MoSe_2_@MXene‐1 cell exhibits the lowest energy consumption value (0.91 kWh kg^−1^‐LiCl). The reduced energy consumption arises from the uniformly dispersed Fe‐THBQ and the robust MoSe_2_@MXene heterostructure, which optimizes interfacial charge distribution, suppresses side reactions, and minimizes polarization losses in the electrode.

To thoroughly assess the Li^+^ capture performance of the Fe‐THBQ@MoSe_2_@MXene‐1 electrode, the influence of initial Li^+^ concentration, operating voltage, and current density on its adsorption behavior was systematically examined. Figure 5c shows the extraction performance of Fe‐THBQ@MoSe_2_@MXene‐1 electrode in different initial LiCl concentrations (10–300 mg L^−1^) at 1.2 V. The results showed that the Li^+^ extraction capacity increased significantly from 1.21 to 19.62 mmol g^−1^ by increasing the solution concentration from 10 to 300 mg L^−1^. This is ascribed to the fact that the higher concentration gradient provides a greater driving force for Li^+^ migration. Figure 5d shows the extraction performance of Fe‐THBQ@MoSe_2_@MXene‐1 electrode in 10 mmol L^−1^ LiCl solution under different applied voltages (0.6–1.4 V). The experimental results showed that the Li^+^ extraction capacity increased first and then decreased by increasing the voltage, and reached a maximum of 4.05 mmol g^−1^ at 1.2 V. Charge efficiency (Λ) is a key indicator to evaluate the effective utilization of charge. When the operating voltage increases from 0.6 to 1.4 V, the charge efficiency decreases from 86% to 63% (Figure 5d; Figure S10). In the lower voltage range (≤ 1.2 V), the input energy is mainly adopted for ion adsorption. However, when the voltage exceeds 1.2 V, the polarization effect of the electrode is enhanced, and the parasitic side reactions (water electrolysis and Cl^−^ oxidation) consume excessive charge. Thus, 1.2 V was selected as the optimal operating voltage. In addition, with increasing the current density, the saturated adsorption capacity increases first and then decreases, reaching a maximum of 3.48 mmol g^−1^ at 20 mA g^−1^ (Figure 5e; Figure S11). This is due to the fact that the high current density strengthens the electric field driving effect and improves the ion migration rate. However, too high current density leads to higher concentration polarization [56].

To elucidate the adsorption mechanism and kinetic behavior of Li^+^ on the Fe‐THBQ@MoSe_2_@MXene‐1 electrode, adsorption isotherms were fitted with pseudo‐first‐order and pseudo‐second‐order kinetic models. In Figure 5g, the electro‐adsorption behavior of Li^+^ on the Fe‐THBQ@MoSe_2_@ MXene‐1 electrode shows a high degree of agreement with the Freundlich isothermal model (R^2^ = 0.99982), which indicates that there are multiple types of adsorption sites on the surface of the material, and multiple adsorption sites cooperate to achieve efficient lithium adsorption. In Figure 5h, Figure S12, and Table S1, the pseudo‐second‐order kinetic model provides a superior fitting effect compared with the pseudo‐first‐order model, suggesting that Li⁺ storage in the electrode material involves not only physical adsorption but also a chemical bonding process [57, 58]. This phenomenon is closely related to the structure of the composite, and the multi‐component cooperation constructs a variety of adsorption sites, and realizes the efficient storage of Li^+^ through the cooperation of redox process and pseudocapacitive intercalation effect. It is noteworthy that the Fe‐THBQ@MoSe_2_@MXene‐1 composite exhibits the lowest pseudo‐second‐order adsorption rate constant (k = 4.04 × 10^−4^), indicating that it has the optimal adsorption performance for Li^+^.

To evaluate the cycling stability, the AC//Fe‐THBQ@MoSe_2_@MXene‐1 cell was subjected to 50 cycles in 5 mmol L‐1 LiCl solution in constant voltage mode (+1.2 V/−1.2 V) (Figure 5i). The results demonstrate that the Fe‐THBQ@MoSe_2_@MXene‐1 electrode retains 91.3% of its initial adsorption capacity after 50 cycles, indicating outstanding durability. This stability is attributed to the superior structural configuration, which effectively mitigates structural degradation during repeated cyclic processes.

To gain insight into the Li^+^ capture mechanism during the CDI process, the electrodes after testing were analyzed by SEM, TEM, XRD, and XPS. In Figure S13a,b,d, SEM, TEM, and HRTEM images of the cycled electrode confirm that its 3D framework structure is completely maintained without noticeable structural collapse or particle agglomeration. This structural stability plays a decisive role in sustaining the cycling performance during CDI testing. The HAADF and the corresponding element distribution map (Figure S13c–j) clearly reveal that Li is uniformly distributed on the surface of the material, which directly confirms the successful capture of Li^+^. In XRD patterns (Figure S13k), the (002) diffraction peak of MoSe_2_ shifted from 13.6° to 11.3°. According to the Bragg equation, the interlayer spacing was significantly enlarged, which confirmed the successful intercalation of Li^+^ into the interlayer structure of MoSe_2_. Meanwhile, the characteristic diffraction peak of Fe‐THBQ at 18.5° shows a marked decrease in intensity, likely resulting from partial dissolution or side reactions with the electrolyte. In addition, the appearance of a new peak at 23.9° suggests the possible formation of chemical bonds between Li^+^ and the electrode material. Additionally, a 1.4° red shift in the (110) plane of MXene suggests lattice expansion of the substrate, resulting from Li⁺ intercalation during the CDI process. XPS analysis of Fe‐THBQ@MoSe_2_@MXene‐1 (Figure S13l) shows that the valence of Fe changes with the intercalation/deintercalation of Li^+^. During the adsorption process, the peak of Fe 2p shifts to lower binding energy, indicating that Fe^2+^ is reduced to Fe_0_. The peak of Fe^2+^ in the peak of Fe 2p weakens, and the peak of Fe_0_ appears. During the desorption process, the peak of Fe_0_ decreased, while the peaks of Fe^2+^ and Fe^3+^ increased, indicating that Fe_0_ lost electrons and was oxidized, and the electropositivity of the composite increased, releasing Li^+^ into the solution.

**FIGURE 6:**
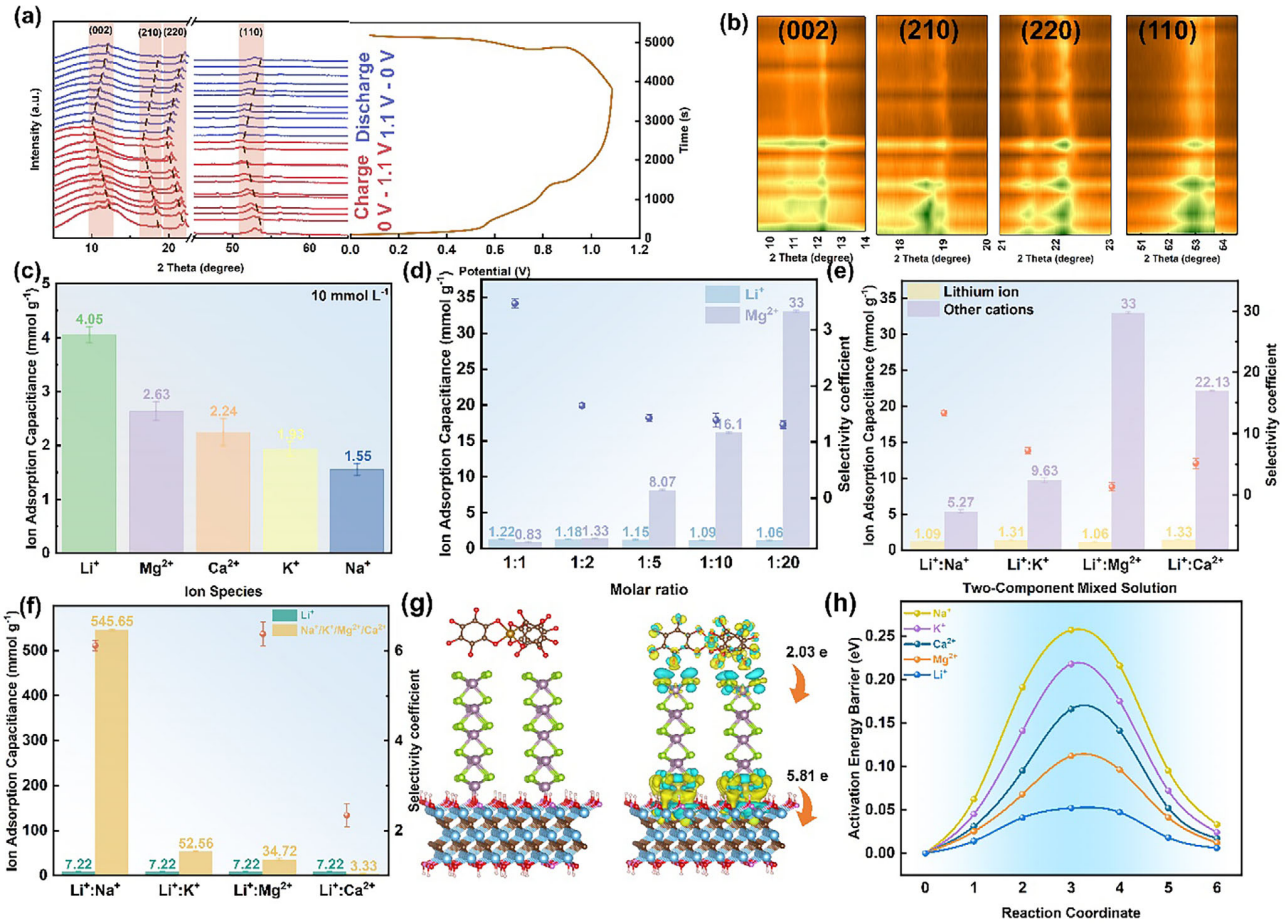
(a) Ex situ XRD analysis and GCD curve of Fe‐THBQ@MoSe_2_@MXene‐1 electrode within 0–1.1 V. b) contour plots of XRD patterns. c) Saturated cation adsorption capacity of AC//Fe‐THBQ@MoSe_2_@MXene‐1 cell in different single salt solutions. d) Cation adsorption capacity and selectivity coefficient of AC//Fe‐THBQ@ MoSe_2_@MXene‐1 battery in mixed solution with different Mg‐Li ratios. e) Cation adsorption capacity and selectivity coefficient of AC//Fe‐THBQ@MoSe_2_@MXene‐1 cell in binary mixed solution. f) Cation adsorption capacity and selectivity coefficient of AC//Fe‐THBQ@MoSe_2_@MXene‐1 cell in real salt lake water. g) The differential charge density of Fe‐THBQ@MoSe_2_@MXene‐1, yellow and blue in the figure, represents electron accumulation and transfer processes. h) The diffusion energy barriers of different cations (Li^+^, Mg^2+^, Ca^2+^, K^+^, Na^+^) in Fe‐THBQ@MoSe_2_@MXene‐1.

In addition, the crystal structure evolution mechanism of Fe‐THBQ@MoSe_2_@MXene‐1 electrode during the electrochemical charge‐discharge process was resolved by ex situ XRD characterization (Figure 6a,b). During the charging process, the (210) and (220) diffraction peaks of Fe‐THBQ show a significant red shift, and the maximum shift occurs when the potential reaches 1.1 V. In the subsequent discharge stage, the positions of the relevant crystal planes are basically restored to the initial state, which fully illustrates that the redox reaction in Fe‐THBQ materials is highly reversible. Furthermore, the (002) and (110) diffraction peaks of MoSe_2_ display a continuous red shift during charging and a corresponding blue shift upon discharging, eventually reverting to their initial positions. This reversible shift behavior directly reveals the dynamic intercalation mechanism of MoSe_2_. During charging, Li^+^ intercalate into the MoSe_2_ interlayers, leading to an expansion of the interplanar spacing. Upon discharging, the subsequent extraction of Li⁺ causes the spacing to contract, ultimately returning to its original state, demonstrating the highly reversible nature of the intercalation process [59]. These findings confirm that the Fe‐THBQ@MoSe_2_@MXene‐1 composite achieves efficient lithium storage via a synergistic dual mechanism combining redox activity and ion intercalation.

The comparative results presented in Table S4 unequivocally position Fe‐THBQ@MoSe_2_@MXene as a highly competitive material, outperforming most traditional materials in both Li^+^ adsorption capacity and rate within comparable testing conditions. This notable advantage stems from the unique material architecture, which successfully integrates the high conductivity of the MoSe_2_@MXene framework with the exceptional Li^+^ adsorption selectivity of the Fe‐THBQ, thereby achieving synergistic enhancement in capacitive deionization performance.

### 2.4 Selective Extraction of Li^+^ and Theoretical Calculation

To evaluate the ion selectivity of the Fe‐THBQ@MoSe_2_@MXene‐1 electrode, a saturation adsorption experiment was first performed in a 10 mmol L^−1^ single solution system (Figure 6c). The results showed that the order of the saturated adsorption amount of each cation was Li^+^ (4.05 mmol g^−1^) > Mg^2+^ (2.63 mmol g^−1^) > Ca^2+^ (2.24 mmol g^−1^) > K^+^ (1.93 mmol g^−1^) > Na^+^ (1.55 mmol g^−1^), confirming that the electrode has a good adsorption effect on Li^+^. In view of the similar hydration radius and chemical properties of Li^+^ and Mg^2+^ in salt lake brine, the efficient separation of Li^+^ and Mg^2+^ is the core problem of lithium extraction in salt lakes. Therefore, the effect of different concentrations of Mg^2+^ on the selective adsorption of Li^+^ in the AC//Fe‐THBQ@MoSe_2_@MXene‐1 cell was studied. Obviously, by increasing Mg^2+^ concentration, the adsorption capacity of Li^+^ decreased, and the Li^+^/Mg^2+^ selectivity coefficient also decreased (Figure 6d). This phenomenon clearly reveals the competition mechanism between Mg^2+^ and Li^+^ in the adsorption sites, and confirms that the presence of Mg^2+^ can significantly interfere with the selective adsorption process of electrode materials for Li^+^. Considering that the actual salt lake brine is a complex multi‐component system containing Li^+^, Mg^2+^, Na^+^, K^+^, Ca^2+,^ and so on, which is difficult to simulate and configure in the laboratory, this study uses binary mixed systems (Li^+^/Mg^2+^, Li^+^/Na^+^, Li^+^/K^+^, Li^+^/Ca^2+^) with controlled variables to carry out systematic research. Accurately analyze the competition mechanism of the target cation to Li^+^. The selectivity of the electrode materials to the target ions follows the rule of Li^+^ > Mg^2+^ > Ca^2+^ > K^+^ > Na^+^ (Figure 6e). The observed selectivity is primarily governed by ionic properties. Li^+^, with the lowest hydration energy, readily undergoes desolvation and binds efficiently to the electrode's active sites. In contrast, although Mg^2+^ and Ca^2+^ possess higher charge densities, their larger hydrated radii create pronounced steric hindrance. However, the adsorption efficiency of K^+^ and Na^+^ in the electrode materials is reduced due to the increase of the desolvation energy barrier caused by their strong hydration despite their smaller hydration radius.

The actual Dachaidan salt lake brine in Qinghai Province (specific composition: Li^+^ 0.07 g L^−1^, Na^+^ 98.56 g L^−1^, K^+^ 2.53 g L^−1^, Mg^2+^ 6.97 g L^−1^, Ca^2+^ 0.42 g L^−1^) was used for verification, and the selectivity order follows: Li^+^ > Ca^2+^ > Na^+^ > Mg^2+^ > K^+^ appears (Figure 6f). This discrepancy is primarily attributed to the formation of [MgSO_4_] complexes between abundant SO_4_2^−^ and Mg^2+^ in the brine, which substantially lowers the concentration of free Mg^2+^. The hydration radius of Ca^2+^ is smaller than that of Mg^2+^, which shows a kinetic advantage and faster diffusion in the pore. These factors together lead to the improvement of the selectivity of Ca^2+^. Notably, the selectivity for Na^+^ increases significantly, primarily due to the strong concentration‐driven force arising from its exceptionally high concentration in the salt lake brine. In addition, K^+^ in salt lake brine has neither the concentration advantage of Na^+^ nor the advantage of charge quantity, which leads to the worst adsorption selectivity. Overall, ion selectivity in real salt lake brines is governed by a combination of concentration effects, complexation, and interfacial interactions, which differs from the behavior observed in laboratory‐simulated solutions. Meanwhile, it also emphasizes the necessity of evaluating materials in realistic environments.

First‐principles calculations based on density functional theory (DFT) reveal the interfacial electron transfer pathways and systematically clarify the selective Li^+^ trapping mechanism in Fe‐THBQ@MoSe_2_@MXene‐1. The differential charge density map (Figure 6g) clearly shows that the electrons are mainly concentrated at the component contact interface, which confirms the strong interface electron coupling. Due to the strong electronegativity of MXene, the electrons present a well‐defined unidirectional transfer path: Fe‐THBQ (electron loss 2.03e) → MoSe_2_ → MXene (electron gain 5.81 e). This directional cascade electron transfer mechanism significantly enhances the interfacial charge separation efficiency and greatly improves the Li^+^ adsorption and reduction ability of the material by optimizing the electron transport kinetics, thus achieving efficient electrochemical lithium extraction performance. Meanwhile, this optimized electronic structure also enhances the conductivity and promotes the electron transfer, significantly lifting the Li^+^ adsorption kinetics, consistent with the EIS results. By simulating the migration paths of different cations and calculating the migration energy of each step (Figure S14; Figure 6h), it can be found that Li^+^ shows the lowest migration energy barrier (0.052 eV), which is significantly lower than Mg^2+^ (0.112 eV), Ca^2+^ (0.166 eV), K^+^ (0.218 eV) and Na^+^ (0.257 eV). This differential migration energy barrier directly determines the adsorption and desorption kinetic behavior of each ion in the electrode, and the lower migration energy barrier not only gives Li^+^ a faster transport rate, but also makes it a competitive advantage in the mixed ion system. These theoretical research results provide a theoretical basis for the excellent Li^+^ selectivity and extraction efficiency of the ternary composite.

## 3 Conclusion

In summary, Fe‐THBQ@MoSe_2_@MXene ternary composites were designed using an innovative heterostructure engineering strategy, achieving efficient adsorption and selective extraction of Li^+^. Specifically, MXene serves as a conductive framework, facilitating efficient electron transport; the layered structure of MoSe_2_ provides rapid diffusion channels for Li^+^, and the abundant pores and active sites of Fe‐THBQ further enhance the Li^+^ adsorption capacity. Moreover, Fe‐THBQ and MoSe_2_@MXene heterointerfaces form Fe─Se coordination bonds, which effectively enhance the interfacial electronic coupling and promote charge transport, thus significantly improving the conductivity and lithium storage kinetics of the materials. The preferred Fe‐THBQ@MoSe_2_@MXene‐1 electrode achieved a high adsorption capacity of 4.05 mmol g^−1^ and a fast adsorption rate of 0.98 mmol g^−1^ min^−1^, exhibiting state‐of‐the‐art extraction performance. Ex situ XRD analysis confirmed that the composite achieves efficient Li^+^ storage through a synergistic mechanism combining redox reaction and ion intercalation. Meanwhile, it maintains high Li^+^ selectivity in different binary mixed solutions and actual Dachaidan salt lake brine. DFT calculation revealed the selective trapping mechanism of Li^+^, showing that its migration energy barrier is significantly lower than that of competing ions, which provided a theoretical basis for efficient lithium extraction from actual salt lake brines.

## 4 Experimental Section

### 4.1 Preparation of the Composite MoSe_2_@MXene

The Al layer was etched by LiF and HCl with Ti_3_AlC_2_ MAX phase as the precursor. Subsequently, the resulting few‐layer MXene nanosheets were centrifuged by a combination of ultrasound and centrifugation. 0.237 g of selenium powder was added to 15 mL of hydrazine hydrate and stirred for 45 min to form solution A. Simultaneously, 0.363 g of sodium molybdate dihydrate and 10 mL of MXene dispersion was added to 40 mL of deionized water, followed by ultrasonic treatment for 45 min to obtain solution B. Solution B was then gradually added to solution A, and the mixture was stirred for an additional 45 min. The mixed solution was transferred to a hydrothermal kettle, and the reaction was carried out at 200°C for 12 h. Upon completion, the product was filtered, washed, and lyophilized, and the final black product obtained was MoSe_2_@MXene.

### 4.2 Preparation of the Composite Fe‐THBQ@MoSe_2_@MXene

4‐Hydroxybenzoquinone (THBQ) was synthesized from 6‐hydroxycyclohexane via oxidation with concentrated nitric acid, followed by precipitation with sodium carbonate (Na_2_CO_3_) to yield sodium tetrahydroxybenzoquinone (Na‐THBQ). 0.21 g of sodium tetrahydroxyquinone (Na‐THQ), 0.1 g of cetyltrimethylammonium bromide (CTAB), and 150 mL of deionized water were added to a flask and stirred until fully dissolved. Subsequently, 0.2 g of MoSe_2_@MXene and 0.28 g of FeSO_4_·7H_2_O were introduced into the solution. The flask was purged with nitrogen gas to maintain an inert atmosphere and then heated in an oil bath at 65°C for 12 h. After the reaction, the system was cooled to room temperature, followed by suction filtration and washing with deionized water three times. The product was cold‐dried for over 12 h to yield the Fe‐THBQ@MoSe_2_@MXene composite. Ternary composite with other ratios was prepared using the same procedure by adjusting the mass ratio of Fe‐THBQ to MoSe_2_@MXene accordingly, denoted Fe‐THBQ@MoSe_2_@MXene‐x (*x* = 0.5, 1, and 1.5), where *x* is the millimolar amount of Fe‐THBQ.

## Conflicts of Interest

The authors declare no conflicts of interest.

## Supporting information




**Supporting File**: advs73685‐sup‐0001‐SuppMat.docx.

## Data Availability

The data that support the findings of this study are available in the supplementary material of this article.
