# Supplementary material for: Heterostructure‐Engineered Conductive MOFs Coupled with MoSe2@MXene Framework for Efficient and Selective Lithium Extraction from Salt Lake Brine via Capacitive Deionization
Source: Adv Sci (Weinh). 2026 Jan 4;13(17):e21830. doi: 10.1002/advs.202521830 (PMC13042383; doi:10.1002/advs.202521830)
Supplement: Supplementary file 1 — Supporting File: advs73685‐sup‐0001‐SuppMat.docx. [file ADVS-13-e21830-s001.docx]

Supporting Information

Heterostructure-engineered Conductive MOFs coupled with MoSe_2_@MXene Framework for Efficient and Selective Lithium Extraction from Salt Lake Brine via Capacitive deionization

Pengze Li^+^, Xiaodan Chong^+^, Yutong Guo, Guangrun Wu, Shanshan Wang, Ruixue Liu, Jiamiao Ma, Yu Wang, Wenqiang Chen, Yanmeng Cai*, Yaoyao Wang*, Qing Yuan*, Jinsheng Zhao*

P. Li, X. Chong, Y. Guo, S. Wang, R. Liu, J. Ma, Y. Cai, Y. Wang, Q. Yuan, J. Zhao

Shandong Key Laboratory of Chemical Energy Storage and Novel Cell Technology, School of Chemistry and Chemical Engineering, Liaocheng University, Liaocheng 252059, China
E-mail: caiyanmeng0315@163.com; [wangyaoyao@lcu.edu.cn](mailto:wangyaoyao@lcu.edu.cn); yuanqing@lcu.edu.cn; [j.s.zhao@163.com](mailto:j.s.zhao@163.com)

G. Wu, Y. Wang

School of Architecture and Civil Engineering, Liaocheng University, Liaocheng 252000, China
W. Chen

Shandong Xinfa Ruijie New Materials Technology Co., Ltd, Liaocheng 252100, China

^+^ P. Li and X. Chong contributed equally to this work.

**Materials characterization**

The morphology, structure, and elemental distribution of the synthesized materials were analyzed using field emission scanning electron microscopy (SEM, 8100, Japan) and transmission electron microscopy (TEM, FEI 200, Japan). The phase and crystal structure of the materials were examined using X-ray diffraction (XRD, Bruker D8-Focus) with a Cu Kα radiation source. Raman spectroscopy (LabRAM HR Evolution, China) was employed to analyze the graphitization degree and defects, while X-ray photoelectron spectroscopy (XPS, ESCALAB-250Xi) was used to investigate surface elemental composition. The specific surface area (SSA) and pore structure of the synthesized materials were determined from N2 adsorption/desorption isotherms using the Brunauer-Emmett-Teller (BET) and Barrett-Joyner-Halenda (BJH) models (Micromeritics Autosorb-iQ2-MP). The concentrations of cations in the mixed solution were monitored using an inductively coupled plasma optical emission spectrophotometer (ICP-OES, Thermo ICS-1100).

**Materials**

Ti_3_AlC_2_ MAX phase (99.9%, 11 Technology Co Ltd), LiF (99.9%, Shanghai Macklin Biochemical Technology Co Ltd), hydrochloric acid (HCl, 38%, Damao Chemical Technology Co Ltd.), Cetyltrimethylammonium bromide (CTAB, Shanghai Aladdin Biochemical Technology Co Ltd), selenium powder (Shanghai Macklin Biochemical Technology Co Ltd), hydrazine hydrate (80%, Liaocheng Tongli Experimental Equipment Co Ltd), Sodium molybdate dihydrate (Shanghai Macklin Biochemical Technology Co Ltd), Ferrous sulfate heptahydrate (FeSO_4_·7H_2_O Shanghai Macklin Biochemical Technology Co Ltd)Polyvinylidene fluoride (PVDF, Dongguan Zhanyang Polymer Material Co Ltd), Graphite powder (Shanghai Aladdin Biochemical Technology Co Ltd), N-methyl pyrrolidone (NMP, Shanghai Aladdin Biochemical Technology Co Ltd), Lithium chloride (LiCl, Shanghai Macklin Biochemical Technology Co Ltd) Sodium chloride (NaCl, Sinopharm Chemical Reagent Co Ltd), potassium chloride (KCl, Sinopharm Chemical Reagent Co Ltd), calcium chloride (CaCl_2_, Sinopharm Chemical Reagent Co Ltd), magnesium chloride (MgCl_2_·6H_2_O, Sinopharm Chemical Reagent Co Ltd) and water. The reagents in the experiment were used directly without further purification.

**Electrochemical measurements**

The calculated specific capacitance (C_s_, F g^-1^) of working electrode was acquired from the Eq. S1:

**$C_{S}=\frac{\int IdV}{2mV\Delta V}$** (S1)

where I (A), V (V) , m (g), v (mV s^-1^) and ΔV (V) refer to the current response, the voltage, the weight of electrode active material, the scan rate the scanning voltage range, respectively.

The discharge specific capacitance (C_v_, F g^-1^) was assessed via the GCD curve from the Eq. S2:

**$C_{V}=\frac{I\Delta t}{m\Delta V}$** (S2)

where I (A), Δt (s), m (g), and ΔV (V) correspond to the discharge current, the discharge time, the weight of electrode active material, and the discharge voltage range, respectively.

**Desalination tests**

Desalination performance indicators including the salt adsorption capacity (SAC, mg g^-1^), the average salt adsorption rate (SAR, mg g^-1^ min^-1^), the charging efficiency (Λ) and the energy consumption (E) which are obtained by the following Eq. S3, Eq. S4, Eq. S5, and Eq. S6.

$SAC=\frac{(C_{0}-C_{e})V}{m}$ (S3)

$SAR=\frac{(C_{0}-C_{e})V}{mt}$ (S4)

$\Lambda=\frac{m\times SAC\times F}{M\int Idt}\times100\%$ (S5)

$E=\frac{U\times\int Idt}{3600(C_{0}-C_{e})V}\times100\%$ (S6)

Where C_0_, C_e_ (mg L^-1^), V (L), m (g), t (s), F (96,485 C mol^-1^), M (58.5 g mol^-1^), U (V) and I (A) correspond to the feed NaCl concentration, the end NaCl concentration at the saturated state, the feed NaCl solution volume, the weight of two electrodes, the electrosorption time, the Faraday's constant, the molar mass of NaCl,

the applied voltage of CDI during charging and the corresponding current, respectively.

Study on selective adsorption performance of working electrode in CDI cell by ion selectivity coefficient. The corresponding Eq. S6 is as follows :

$\beta=(\frac{M}{Na^{+}})=(\frac{C_{M,initial}-C_{M,final}}{C_{Na^{+},initial}-C_{Na^{+},final}})(\frac{C_{Na^{+},initial}}{C_{M,initial}})$ (S7)

where M correspond to K^+^, Ca^2+^, Mg^2+^; $C_{M,initial}$ and $C_{M,final}$ (mg L^-1^) correspond to the concentration of initial cation M and concentration of saturated cation M in the mixed solution, and (mg L^-1^) correspond to the concentration of initial Na^+^ and concentration of saturated Na^+^ in the mixed solution.

**The electrosorption isotherm model**

The electrosorption isotherm of the three cells with different initial NaCl concentrations is investigated by the Langmuir and Freundlich isotherms models. The Langmuir and Freundlich equations are displayed in the following:

$q_{e}=\frac{q_{m}K_{L}C_{e}}{1+K_{L}C_{e}}$ (S8)

$q_{e}=K_{F}C_{e}^{\frac{1}{n}}$ (S9)

where Ce (mg L^-1^) stands for the equilibrium concentration and qe (mg g^-1^) is the adsorption capacity of the cell, respectively; qm (mg g^-1^) represents the maximum adsorption amount; K_L_ and K_F_ correspond to the Langmuir and Freundlich model constants, respectively; 1/n is the characteristic factor that derives from the linear adsorption.

**Density functional theory calculation**

The density functional theory (DFT) calculations were carried out with the VASP code. The Perdew-Burke-Ernzerhof (PBE) functional within generalized gradient approximation (GGA) was used to process the exchange-correlation, while the projectoraugmented-wave pseudopotential (PAW) was applied with a kinetic energy cut-off of 500 eV, which was utilized to describe the expansion of the electronic eigenfunctions. The Brillouin-zone integration was sampled by a Γ-centered 5 × 5 × 1 Monkhorst-Pack k-point. All atomic positions were fully relaxed until energy and force reached a tolerance of 1 × 10^-6^ eV and 0.01 eV/Å, respectively. The dispersion corrected DFT-D method was employed to consider the long-range interactions.


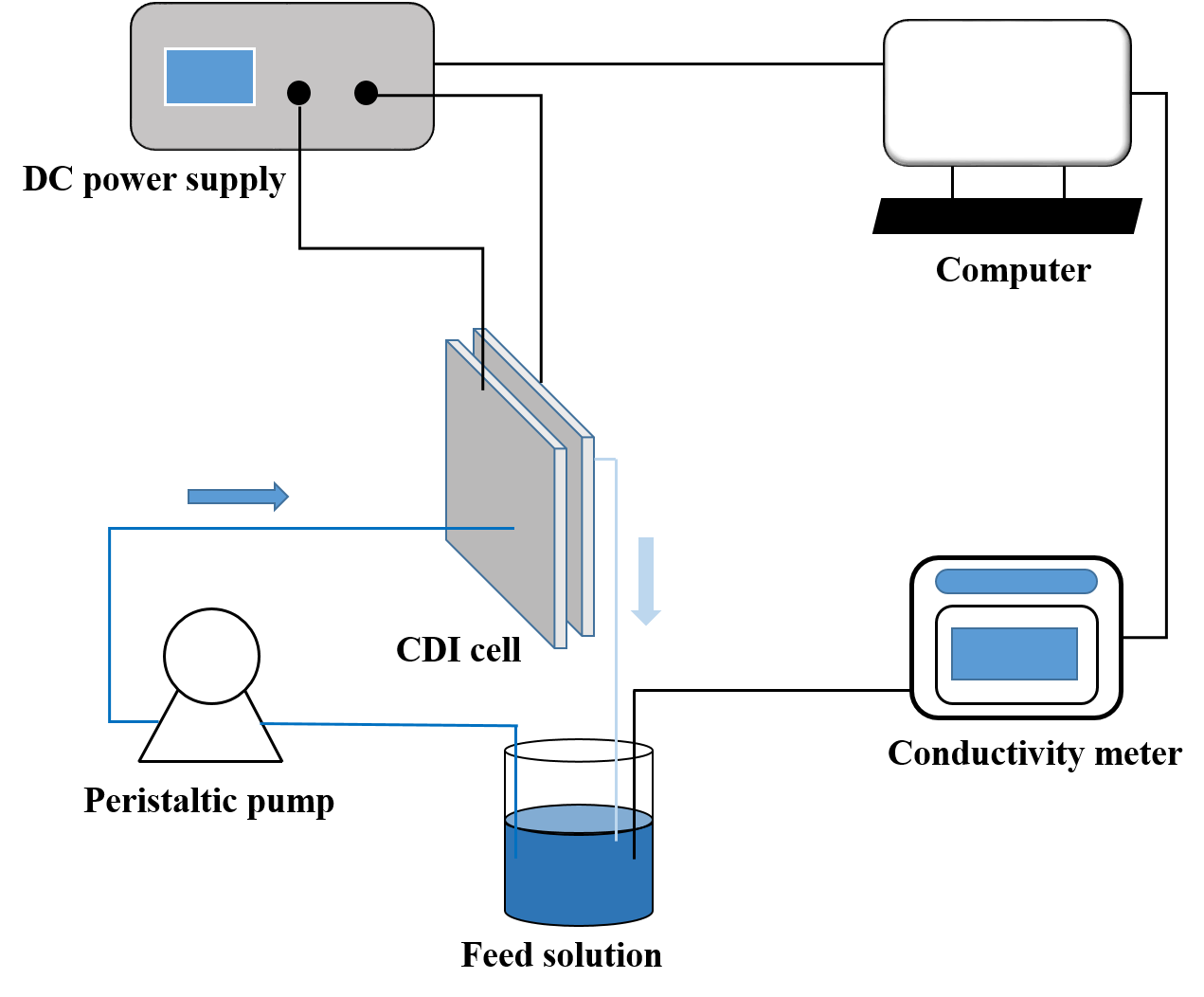


**Figure S1.** The schematic illustration of the desalination setup for CDI process.

**
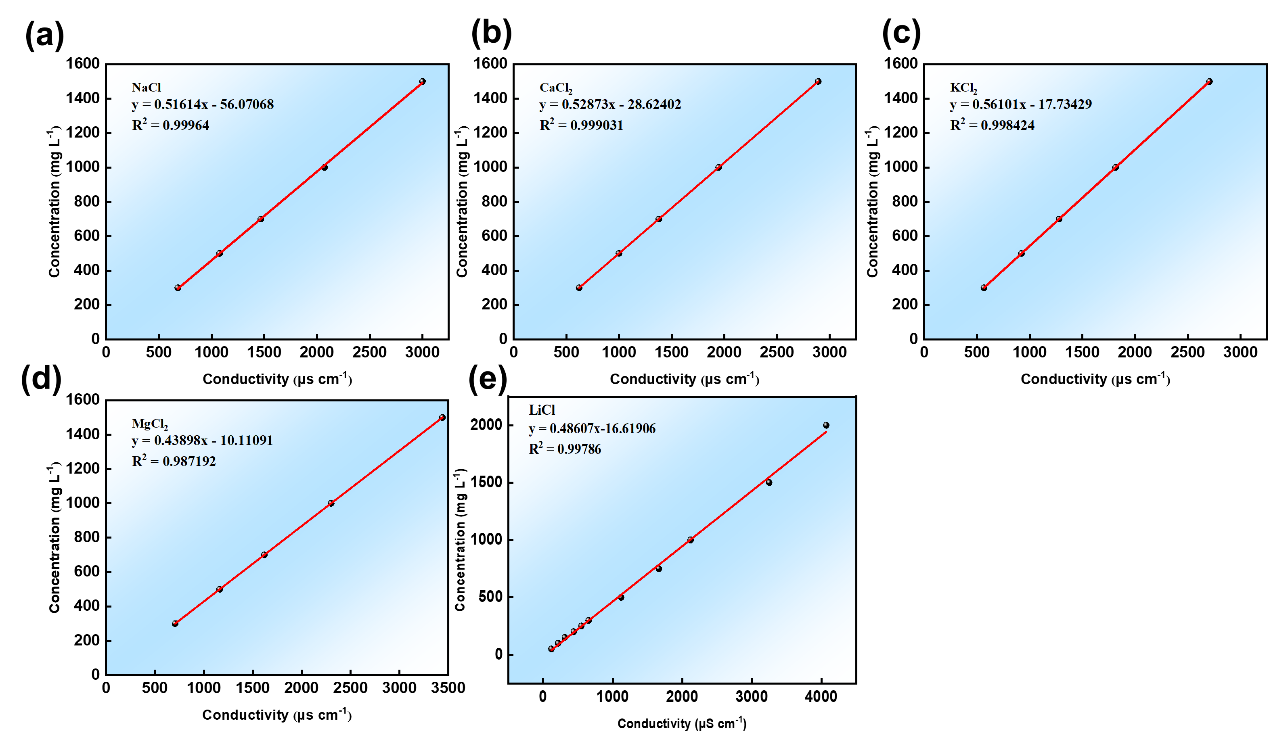
**

**Figure S2.** Calibration curve for the concentration-conductivity relationship of a) NaCl, b) CaCl_2_,c) KCl, d) MgCl_2_, and e) LiCl solution.


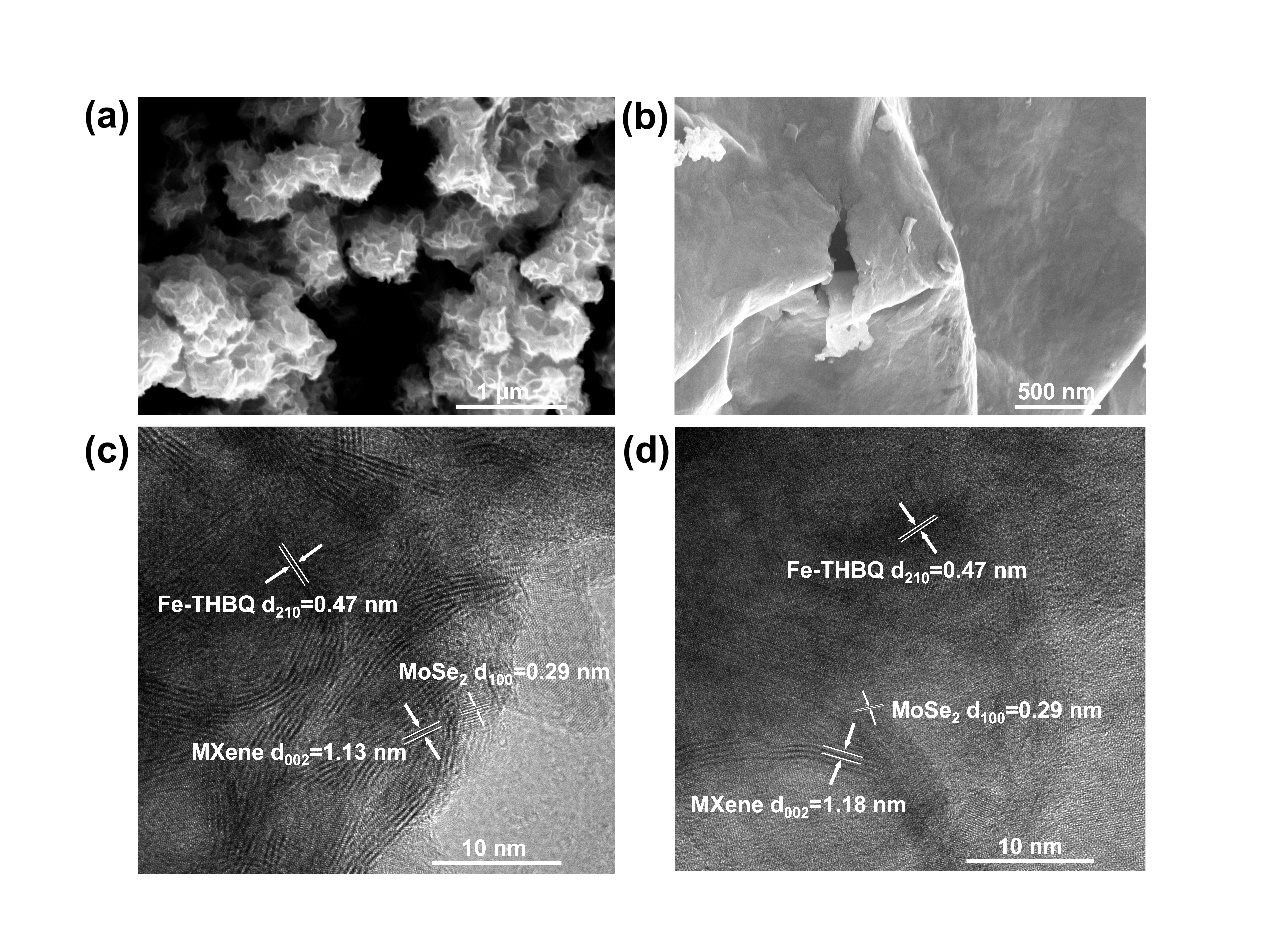


**Figure S3.** SEM images of a) pure MoSe_2_, b) MXene, c, d) HRTEM of Fe-THBQ@MoSe_2_@MXene-1.


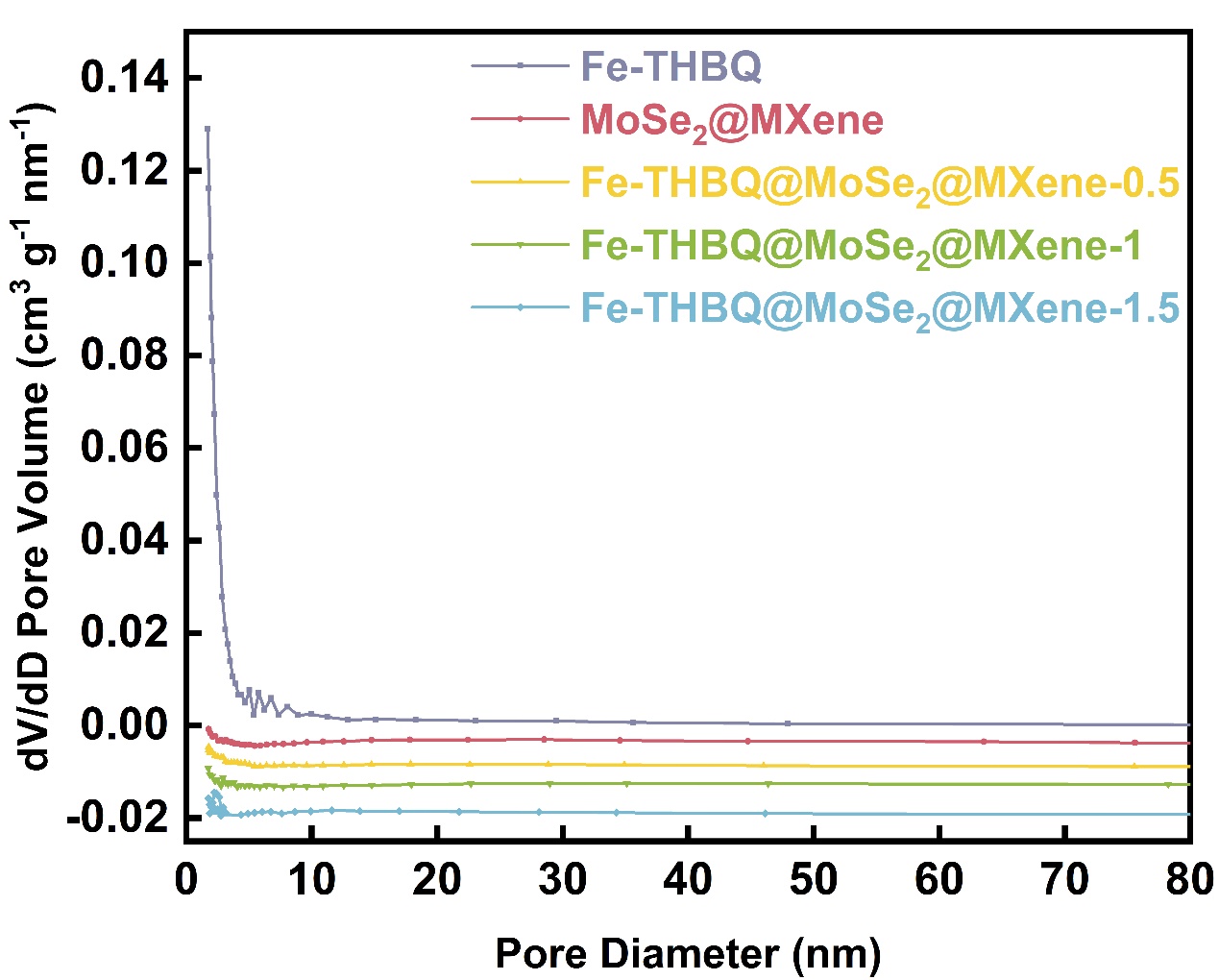


**Figure S4.** The pore size distribution of five materials.

**Figure S5.** High-resolution XPS spectra of Fe-THBQ@MoSe_2_@MXene-1: O 1s.


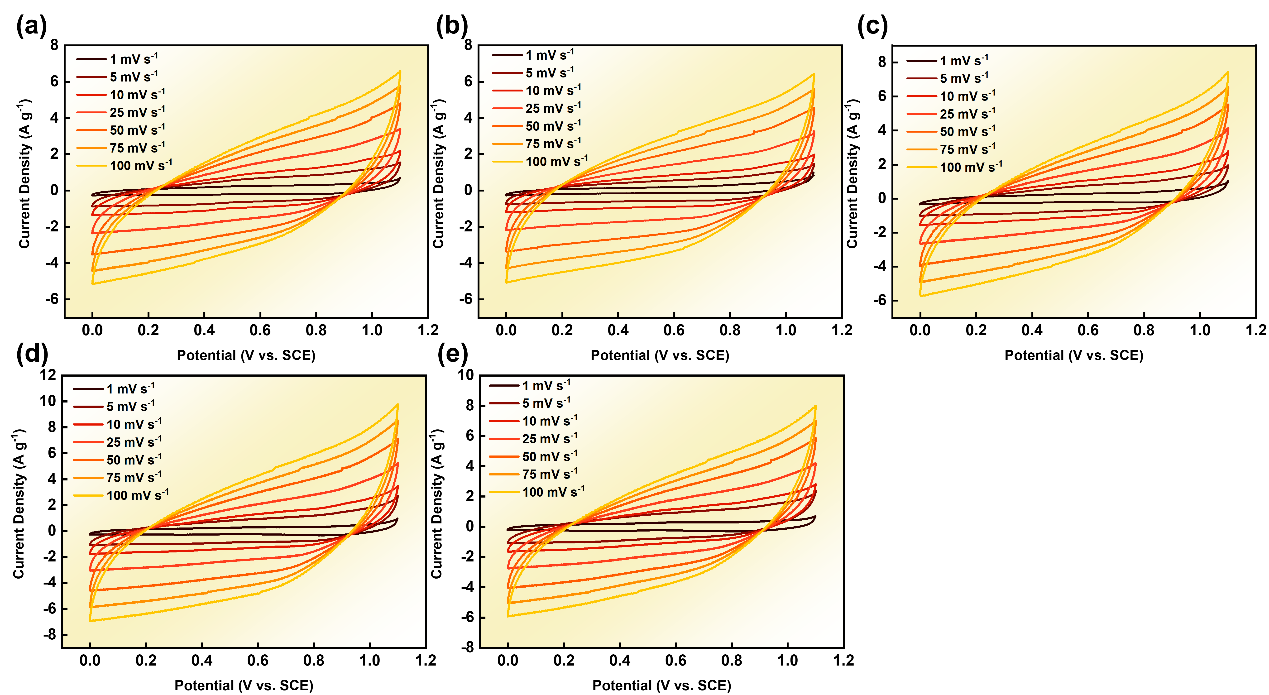


**Figure S6.** CV curves of a) Fe-THBQ, b) MoSe_2_@MXene, c-e) Fe-THBQ@MoSe_2_@MXene-x (x = 0.5, 1, 1.5) at different scan rates (1-100 mV s^-1^).

**
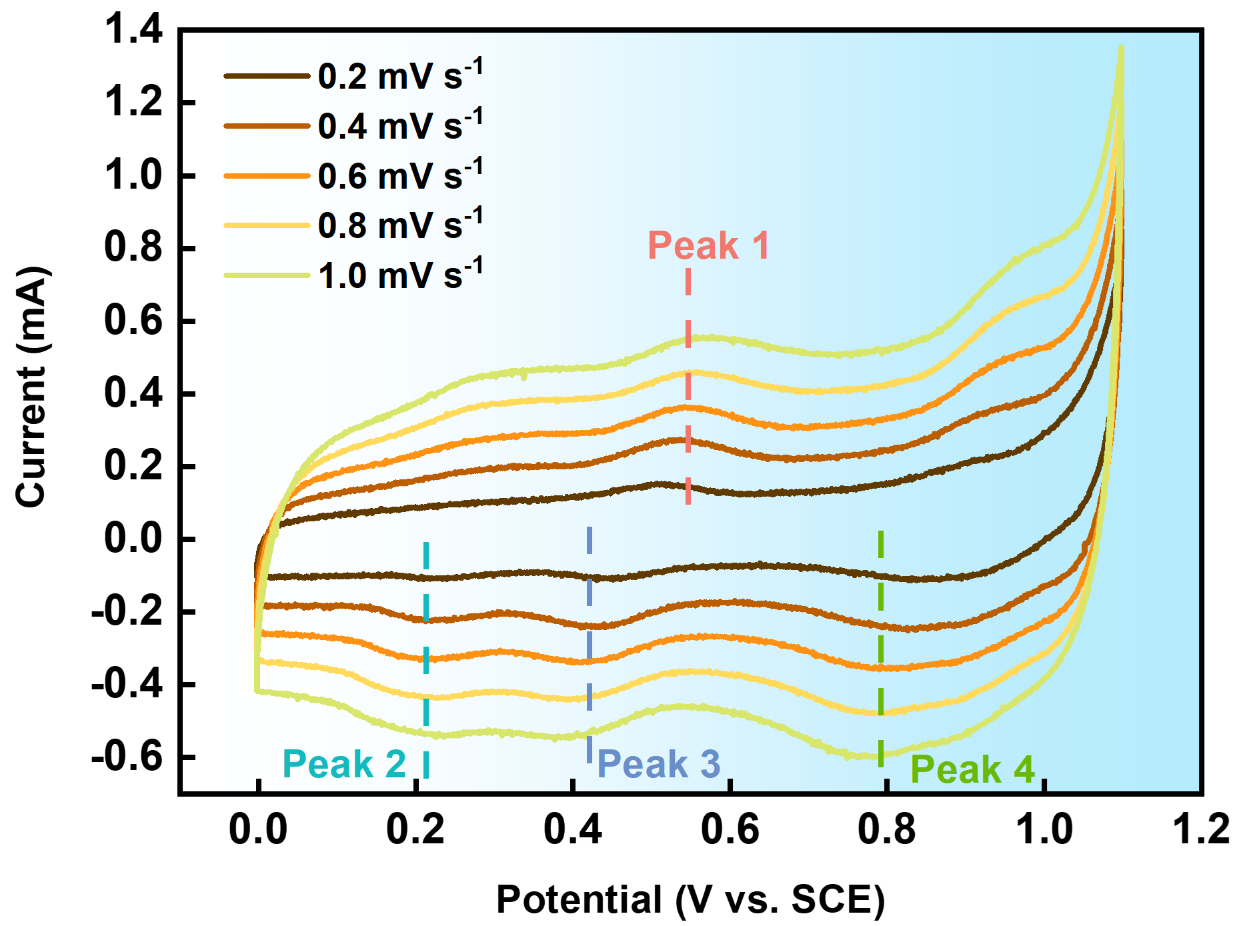
**

**Figure S7.** CV curves of Fe-THBQ@MoSe_2_@MXene-1 at different scan rates (0.2-1.0 mV s-1).

**
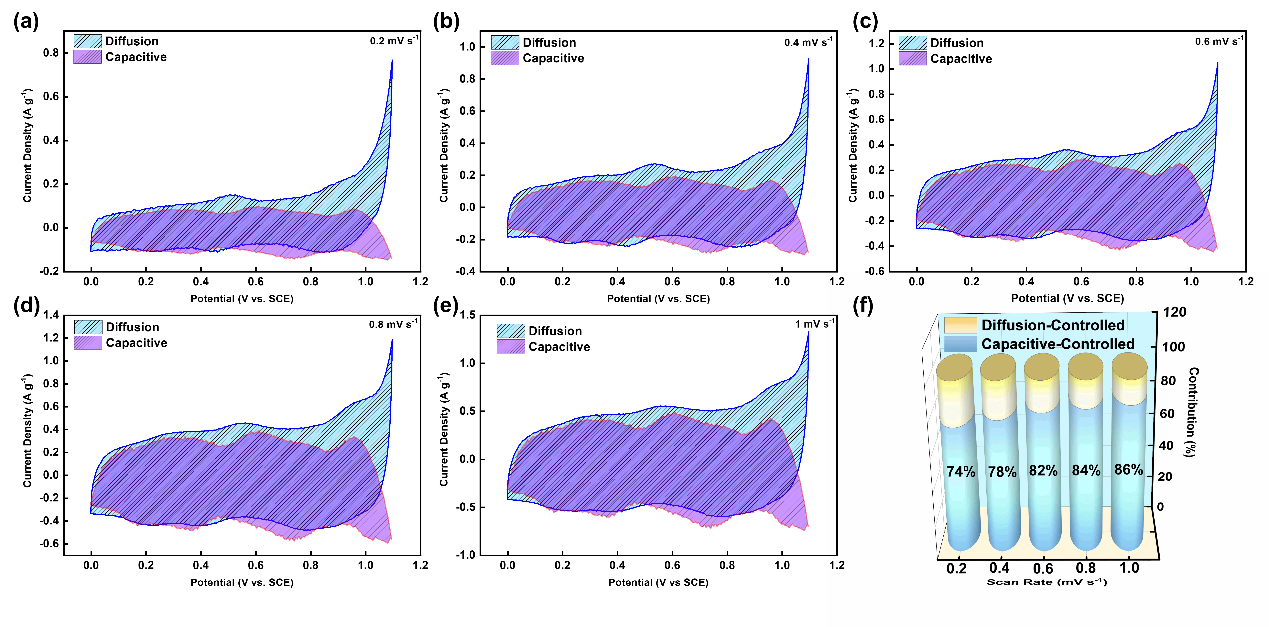
**

**Figure S8.** Proportion of diffusion-controlled and capacitive-controlled Fe-THBQ@MoSe_2_@MXene -1 at different scan rates (0.2-1.0 mV s^-1^).

**
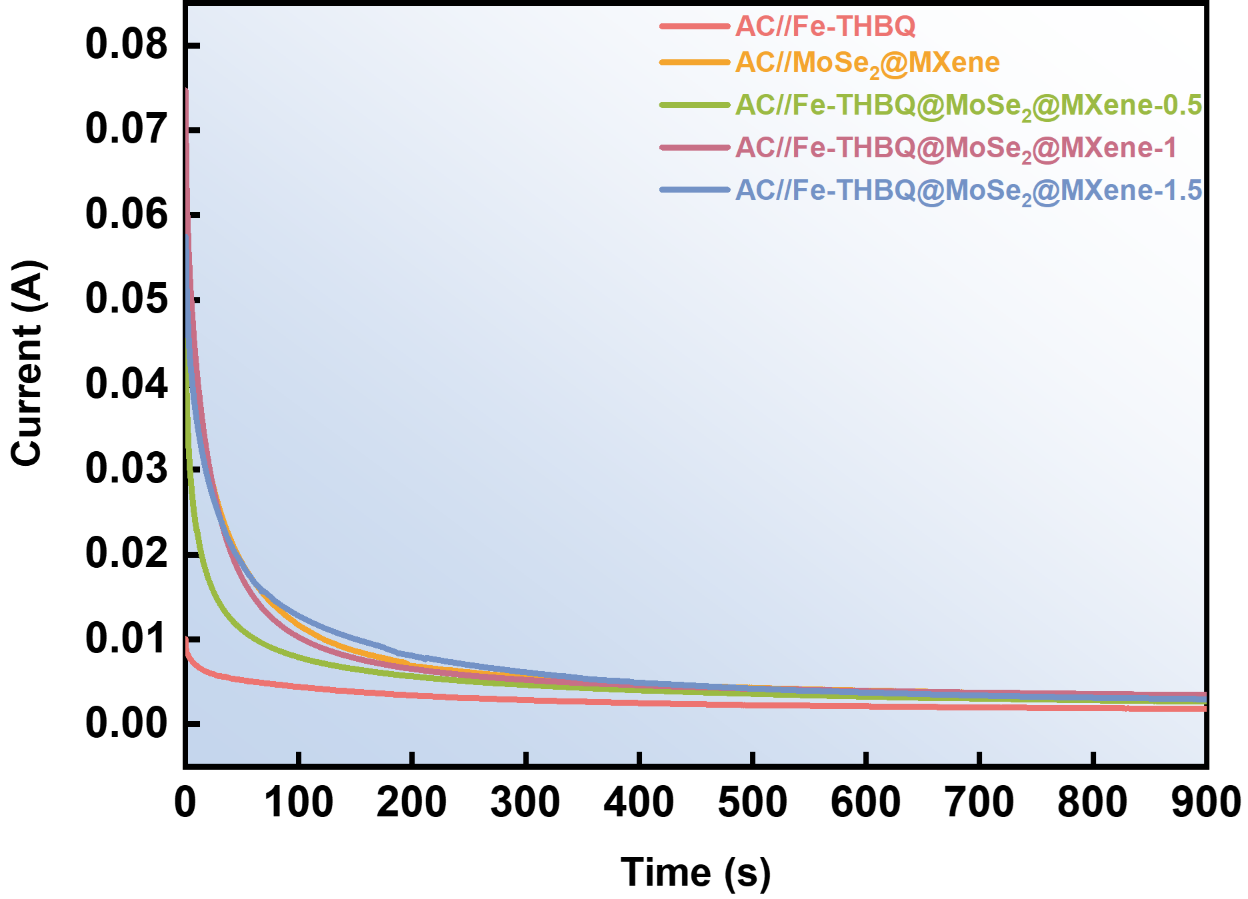
**

**Figure S9.** Current-time curves during the adsorption of five modules.


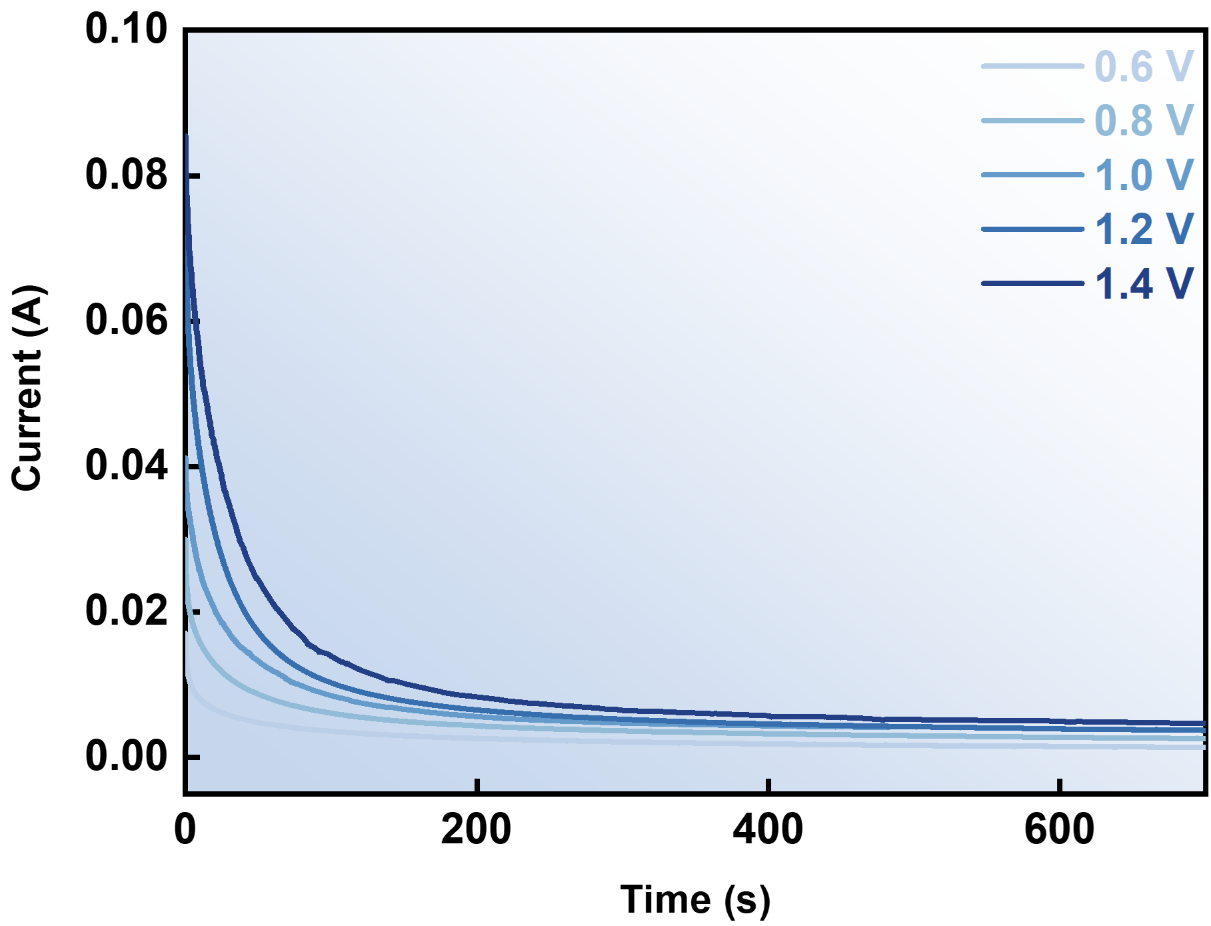


**Figure S10.** Current-time curves of AC//Fe-THBQ@MoSe_2_@MXene-1 cell at different voltages.

**
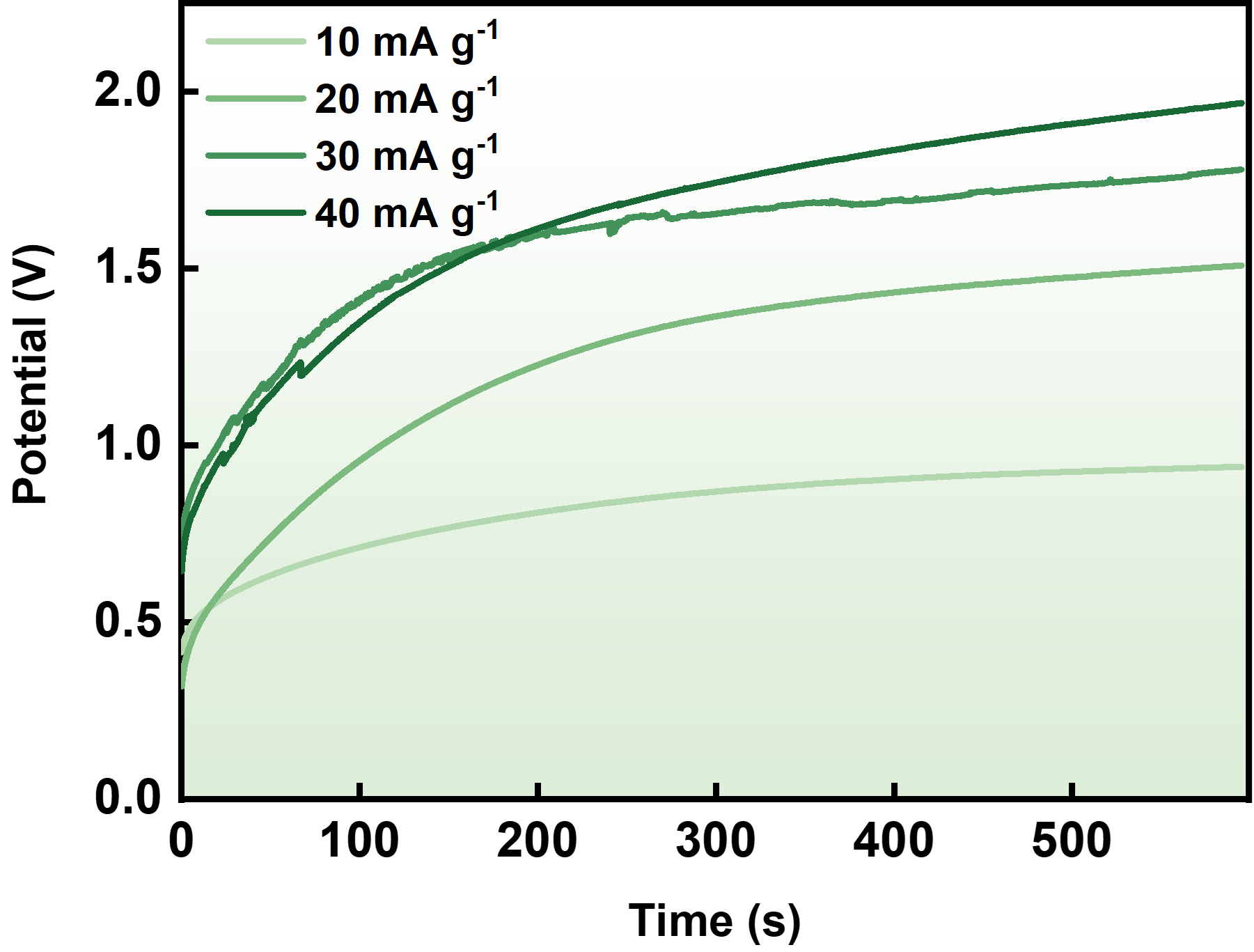
**

**Figure S11.** Potential-time curves of AC//Fe-THBQ@MoSe_2_@MXene-1 cell at different current density.

**
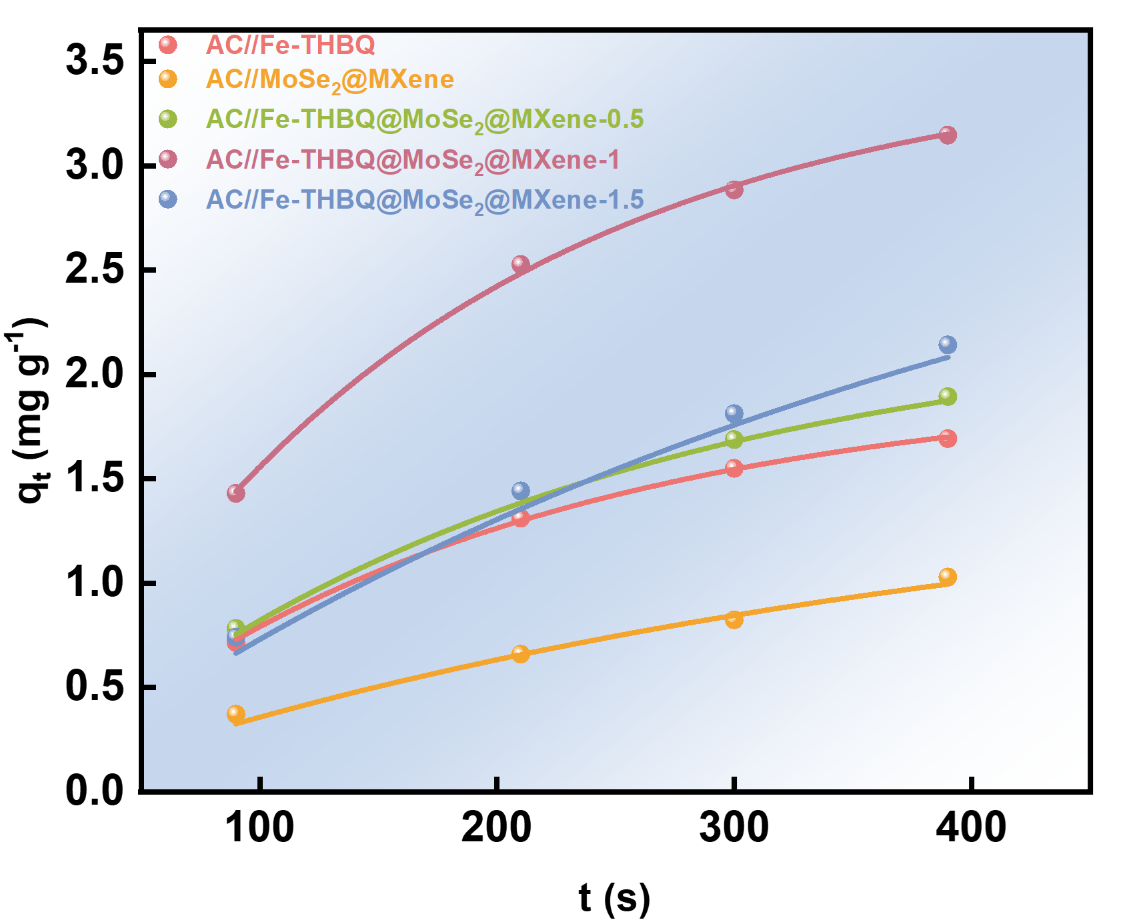
**

**Figure S12.** The pseudo-first-order kinetic fitting curves for five CDI cells.


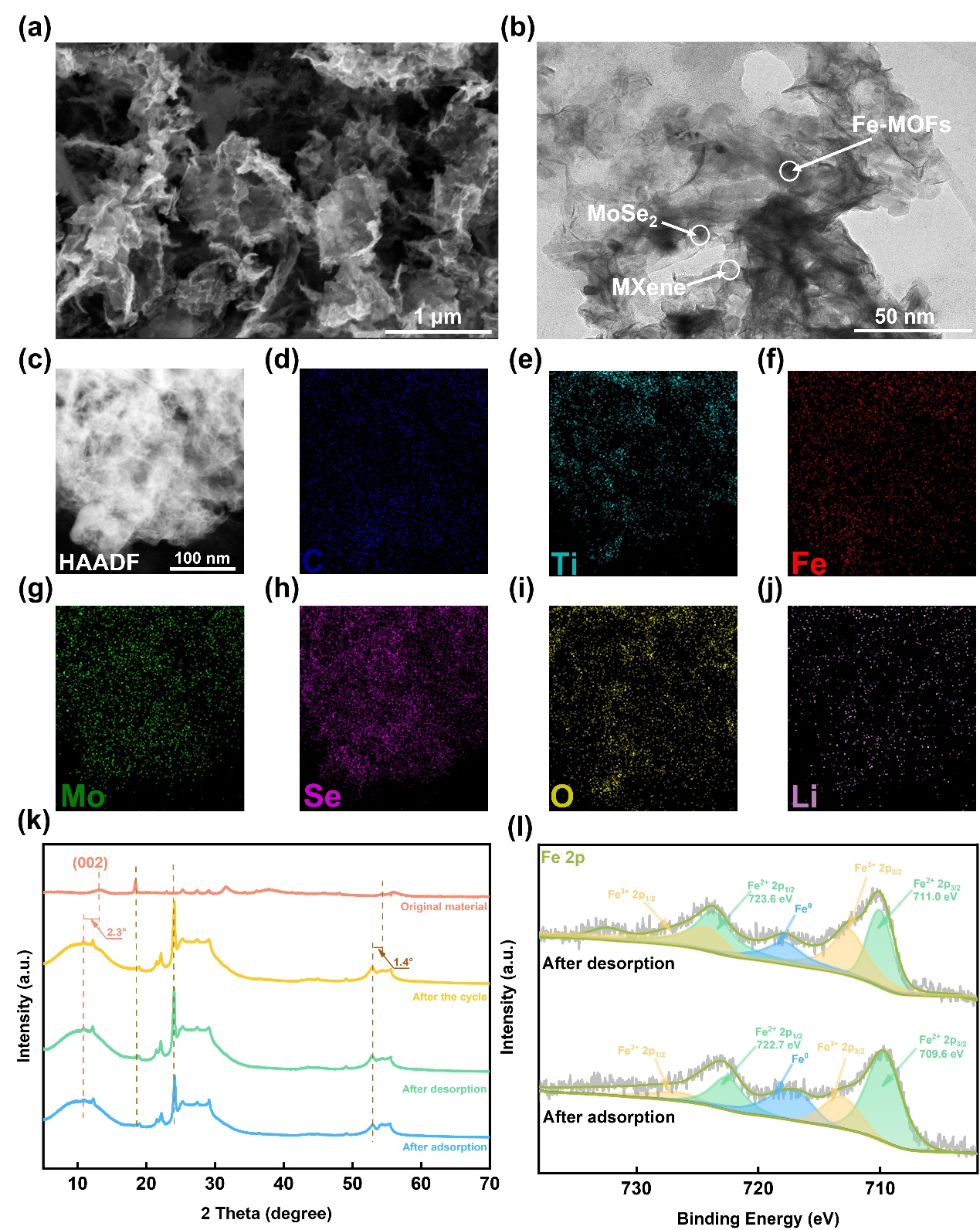


**Figure S13.** a) The SEM image, b) the TEM image, c-j) the HAADF-STEM image and corresponding elemental mappings of Fe-THBQ@MoSe_2_@MXene-1 after Li^+^ adsorption. k) XRD patterns of Fe-THBQ@MoSe_2_@MXene-1 electrode after saturation adsorption, desorption, and cycling. l) High-resolution XPS Fe 2p spectra of Fe-THBQ@MoSe_2_@MXene-1 after saturation adsorption and desorption.


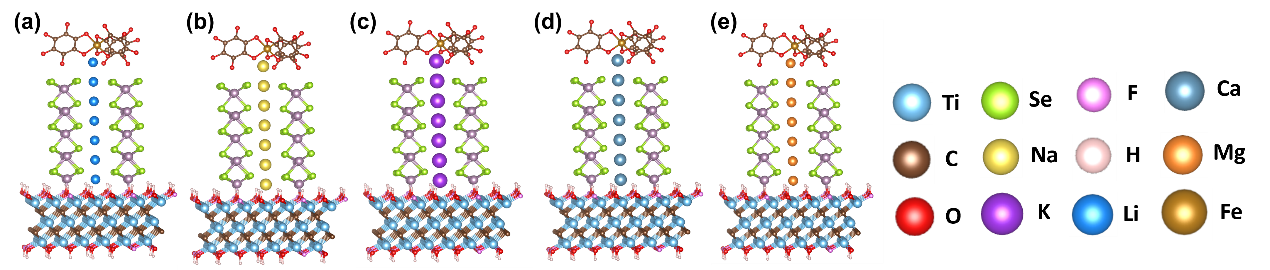


**Figure S14.** Simulation of Diffusion Pathways of Different Cations in Fe-THBQ@ MoSe_2_@MXene-1.

**Table S1.** The electrosorption rate constant *k* and regression coefficient R^2^ of the pseudo-first-order kinetic and pseudo-second-order kinetics for the fire cells

| **Kinetic model** |  | | **Cell name** | | | | |
| --- | --- | --- | --- | --- | --- | --- | --- |
|  | Parameter | AC//Fe-THBQ | AC//MoSe_2_@MXene | AC//Fe-THBQ@MoSe_2_@MXene-0.5 | AC//Fe-THBQ@MoSe_2_@MXene-1 | AC//Fe-  THBQ@MoSe_2_  @MXene-1.5 |  |
| pseudo-first-order | *k*(s^-1^)  R^2^ | 2.16×10^-4^  0.99884 | 1.86×10^-3^  0.97603 | 2.28×10^-3^  0.99024 | 1.15×10^-3^  0.99798 | 9.81×10^-3^  0.97281 |  |
| pseudo-second order | *k*(s^-1^)  R^2^ | 1.33×10^-3^  0.99269 | 1.02×10^-3^  0.94888 | 1.1×10^-4^  0.99536 | 4.04×10^-4^  0.99832 | 9.80×10^-4^  0.99861 |  |

**Table S2.** Physical and thermodynamic properties of the cations in this study.

| **Cation** | **Diffusion coefficient**  **(10^-9^ m^2^ s^-1^)** | **Ion Radius**  **(Å)** | **Hydrated Radius**  **(Å)** | **Hydration free**  **energy (kJ mol^-1^)** |
| --- | --- | --- | --- | --- |
| Na^+^ | 1.33 | 0.95 | 3.58 | -365 |
| K^+^ | 1.96 | 1.33 | 3.31 | -295 |
| Mg^2+^ | 0.71 | 0.72 | 4.28 | -1830 |
| Ca^2+^ | 0.79 | 0.99 | 4.12 | -1505 |
| Li^+^ | 1.00 | 0.76 | 3.82 | -470 |

**Table S3.** Actual content of Fe-THBQ in composites determined by ICP-OES.

| **Sample** | **Fe Element Content**  **(wt%)** | **Fe-THBQ**  **Content**  **(wt%)** |
| --- | --- | --- |
| Fe-THBQ@MoSe_2_@MXene-0.5 | 7.58 | 30.39 |
| Fe-THBQ@MoSe_2_@MXene-1 | 13.69 | 54.89 |
| Fe-THBQ@MoSe_2_@MXene-1.5 | 16.81 | 67.39 |

**Table S4.** Quantitative comparison between this work and other materials

| **Material** | **Li^+^**  **Concentration**  **(mmol L^-1^)** | **Li^+^ Adsorption Capacity**  **(mmol g^-1^)** | **Li^+^**  **Adsorption Rate**  **(mmol g^-1^ min^-1^)** | **Cycle Stability**  **(%)** | **Selectivity**  **Coefficient**  $\boldsymbol{\alpha}_{\boldsymbol{Mg}^{\boldsymbol{2+}}}^{\boldsymbol{Li}^{\boldsymbol{+}}}$ | **References** |
| --- | --- | --- | --- | --- | --- | --- |
| CoAl-LDO | 10 | 1.35 | 0.57 | 70.83%  (20 cycles) | 6.09 | ^[1]^ |
| LMO/LAO | 5 | 0.9 | 0.0324 | 90.00%  (20 cycles) | 5.24 | ^[2]^ |
| LVO/rGO | 15 | 5.69 | 0.393 | 78.02%  (20 cycles) | - | ^[3]^ |
| AlF_3_@LMO | 3.54 | 0.74 | 0.1 | 80.00%  (10 cycles) | 3.67 | ^[4]^ |
| rGO/LMO | 50 | 4.34 | 0.33 | 90.73%  (50 cycles) | 3.22 | ^[5]^ |
| δ-MnO_2_-x@CNTs | 10 | 1.014 | 0.122 | 71.90%  (9 cycles) | - | ^[6]^ |
| Li_2_TiO_3_ | 10 | 1.97 | 0.058 | 96.32%  (50 cycles) | - | ^[7]^ |
| AC/PB | 5 | 3.52 | 0.39 | 95.11%  (50 cycles) | - | ^[8]^ |
| LMTO | 20 | 2.02 | 1.04 | - | 2.14 | ^[9]^ |
| LiO-FeO-Mn_2_O_3_ | 20 | 1.3 | 0.08 | 93.60%  (30 cycles) | - | ^[10]^ |
| Fe-THBQ@MoSe_2_  @MXene | 10 | 4.05 | 0.98 | 92.1%  (35 cycles) | 3.7 | This work |

**Reference:**

[1] Y. Qiao, Y. Li, C. Wang, Q. Pan, B. Chen, Y. Wang, *ACS Sustainable Chemistry & Engineering* **2024**, *12*, 11692.

[2] T. Elmakki, S. Zavahir, H. K. Shon, G. H. Gago, H. Park, D. S. Han, *Desalination* **2025**, *593*, 118195.

[3] X. Shang, Z. Liu, W. Ji, H. Li, *Separation and Purification Technology* **2021**, *262*, 118294.

[4] J. Li, L. Han, R. Wang, T. Wang, L. Pan, X. Zhang, C. Wang, *Desalination* **2024**, *591*, 118035.

[5] H. Zhang, Z. Huang, L. Zhao, Z. Guo, J. Wang, J. Liu, Y. Zhao, F. Li, P. Zhang, Z.-Y. Ji, *Chemical Engineering Journal* **2024**, *482*, 148802.

[6] J. Si, C. Xue, S. Li, L. Yang, W. Li, J. Yang, J. Lan, N. Sun, *Desalination* **2024**, *572*, 117154.

[7] G. Bhaskaran, M. Rethinasabapathy, J. Shin, K. S. Ranjith, H. U. Lee, W. K. Son, Y.-K. Han, T. Ryu, Y. S. Huh, *Journal of Colloid and Interface Science* **2023**, *650*, 752.

[8] M. Rethinasabapathy, G. Bhaskaran, S.-K. Hwang, T. Ryu, Y. S. Huh, *Chemosphere* **2023**, *336*, 139256.

[9] A. Siekierka, *Desalination* **2022**, *527*, 115569.

[10] A. Siekierka, *Separation and Purification Technology* **2020**, *236*, 116234.
